# Supplementary material for: Antiquity and fundamental processes of the antler cycle in Cervidae (Mammalia)
Source: Naturwissenschaften. 2020 Dec 16;108(1):3. doi: 10.1007/s00114-020-01713-x (PMC7744388; doi:10.1007/s00114-020-01713-x)
Supplement: Supplementary file 1 — (PDF 91.8 kb) [file 114_2020_1713_MOESM1_ESM.pdf]

Online Resource 1. Specimens investigated. NMA = Naturmuseum Augsburg, NMB = Naturhistorisches Museum Basel, SMNS = Staatliches Museum für Naturkunde Stuttgart, SNSB – BSPG = Staatliche Naturwissenschaftliche Sammlungen Bayerns – Bayerische Staatssammlung für Paläontologie und Geologie, SNSB – ZSM = Staatliche Naturwissenschaftliche Sammlungen Bayerns – Zoologische Staatssammlung München. For biochronological assessment of sites of provenance see de Bruijn et al. (1992) and for geographical location Thenius (1959).

**Catalogue and Figure Number**	**Species Identification**	**Site of Provenance**	**Geological Age**	**Specimen**	**Method of Examination**	**Literature Presence**
SNSB-ZSM 1966 237b (Online Resource 37)	*Muntiacus muntjak* (Zimmerman, 1780)	zoo specimen, Tierpark Hellabrunn München, Germany	Recent	Shed antler	µCT (110 kV, 0.05 mA, 0.1 mm Cu filter, 1400 images, voxel size 27.5 µm)	Heckeberg (2017b: Figs 1G-I)
SNSB-BSPG 1950 I 30 (Fig. 5j-k, Online Resource 36)	*Euprox furcatus* (Hensel, 1859)	Massenhausen, Germany	Middle Miocene, MN8	Attached antler	µCT (120 kV, 0.06 mA, 0.2 mm Cu filter, 1440 images, voxel size 68.1 µm)	Gentry et al. (1999: Fig. 23.13)
SNSB-BSPG 1966 XIV 34 (Online Resource 35)	*Euprox furcatus* (Hensel, 1859)	Breitenbrunn, Germany	Middle Miocene, MN8	Shed antler	µCT (120 kV, 0.06 mA, 0.2 mm Cu filter, 1440 images, voxel size 51.4 µm)	Gentry et al. (1999: Fig. 23.13); Heckeberg (2017b: Figs 6E-F)
NMB Sth. 12 (Fig. 7, Online Resources 2, 3, 9)	*Euprox furcatus* (Hensel, 1859)	Steinheim, Germany	Middle Miocene, MN7	pedicle without antlers	thin-sections	
SMNS no number (Online Resource 34)	*Heteroprox larteti* (Filhol, 1890)	Steinheim, Germany	Middle Miocene, MN7	skull with attached antlers	µCT (160 kV, 0.07 mA, 1440 images, voxel size 66.7 µm)	
NMA 79-5004/761 **holotype** (Fig. 8a-b, Online Resource 33)	*Paradicrocerus elegantulus* (Roger, 1898)	Stätzling, Germany	Middle Miocene, MN6	Attached antler	µCT (130 kV, 0.037 mA, 0.2 mm Cu filter, 1400 images, voxel size 66.7 µm)	Roger (1904: pl. III, fig. 1); Stehlin (1937: Fig. 1); Azanza and Menéndez (1990: Fig. 3.3)
SNSB-BSPG 1976 VI 24 (Fig. 5c-f, Online Resources 2, 3, 13)	*Paradicrocerus elegantulus* (Roger, 1898)	Thannhausen, Germany	Middle Miocene, MN6	Shed antler	thin-sections	
SNSB-BSPG 1993 I 21 (Online Resource 32)	*Paradicrocerus elegantulus* (Roger, 1898)	Wollersdorf, Germany	Middle Miocene, MN5	Shed antler	µCT (120 kV, 0.06 mA, 0.2 mm Cu filter, 1440 images, voxel size 51.6 µm)	Heckeberg (2017b: Figs 5E-F)
SNSB-BSPG 1993 I 35 (Online Resource 31)	*Dicrocerus elegans* Lartet, 1837	Sansan, France	Middle Miocene, MN6	Attached antler	µ-CT (130 kV, 0.060 mA, 0.2 mm Cu filter, 1400 images, voxel size 54.6 µm)	Gentry et al. (1999: Fig 23.12)
NMB San. 15061 (Online Resources 2, 3, 12)	*Dicrocerus elegans* Lartet, 1837	Sansan, France	Middle Miocene, MN6	Tine, proximal portion	thin-sections	
NMB San. 15062 (Online Resource 2, 3, 12)	*Dicrocerus elegans* Lartet, 1837	Sansan, France	Middle Miocene, MN6	Tine, distal portion	thin sections	

SNSB-BSPG 1959 II 678 (Online Resource 30)	*Lagomeryx parvulus* Roger, 1898	Sandelzhausen, Germany	Middle Miocene, MN5	attached antler	µCT (120 kV, 0.06 mA, 0.1 mm Cu filter, 1438 images, voxel size 11.4 µm)	Fahlbusch (1977: Pl. 16 Figs 1-7); Rössner (2010: Fig. 8Y)
SNSB-BSPG 1959 II 4594 (Online Resources 2, 3, 10, 11)	*Lagomeryx parvulus* Roger, 1898	Sandelzhausen, Germany	Middle Miocene, MN5	Attached antler	thin-sections	Rössner (2010: Figs 8A, G)
SNSB-BSPG 1959 II 12314 (Online Resource 2, 3, 4, 8)	*Heteroprox eggeri* Rössner, 2010	Sandelzhausen, Germany	Middle Miocene, MN5	Attached antler	thin-sections	
SNSB-BSPG 1959 II 5270 (Fig. 4, Online Resource 2, 3, 4, 7)	*Heteroprox eggeri* Rössner, 2010	Sandelzhausen, Germany	Middle Miocene, MN5	Shed antler	thin-sections	
SNSB-BSPG 1959 II 5249 **holotype** (Online Resource 29)	*Heteroprox eggeri* Rössner, 2010	Sandelzhausen, Germany	Middle Miocene, MN5	Attached antler, fully grown, tips missing	µCT (120 kV, 0.06 mA, 0.1 mm Cu filter, 1650 images, voxel size 52.3 µm)	Rössner (2010: Fig. 6A)
SNSB-BSPG 1959 II 2502 (Online Resource 28)	*Heteroprox eggeri* Rössner, 2010	Sandelzhausen, Germany	Middle Miocene, MN5	Attached antler; unbranched, ?first generation	µCT (120 kV, 60 µA, 0.1 mm Cu filter, 1650 images, voxel size 45.4 µm)	Rössner (2010: Fig. 6B)
SNSB-BSPG 1959 II 5258 (Fig. 5g-i, Online Resource 27)	*Heteroprox eggeri* Rössner, 2010	Sandelzhausen, Germany	Middle Miocene, MN5	Shed antler, base preserved only	µCT (120 kV, 60 µA, 0.1 mm Cu filter, 1400 images, voxel size 27.1 µm)	Rössner (2010: Fig. 6E); Heckeberg (2017b: Figs 2E-F)
SNSB-BSPG 1959 II 5268 (Fig. 6b, Online Resource 26)	*Heteroprox eggeri* Rössner, 2010	Sandelzhausen, Germany	Middle Miocene, MN5	Shed antler, base preserved only	µCT (130 kV, 30 µA, 0.1 mm Cu filter, 1400 images, voxel size 25.8 µm)	Rössner (2010: Fig. 6C)
SNSB-BSPG 1976 XXI 64 (Online Resource 25)	*Procervulus dichotomus* (Gervais, 1849)	Langenau 2, Germany	Early Miocene, MN4	Shed antler	µCT (120 kV, 50 µA, 0.1 mm Cu filter, 1700 images, voxel size 33.3 µm)	Heckeberg (2017b: Figs 3K-L)
SMNS 45140 (Online Resource 38)	*Procervulus dichotomus* (Gervais, 1849)	Langenau, Germany	Early Miocene, MN4	Skull with left and right pedicle bud and dentition with M3 erupted but unworn	µCT (130 kV, 30 µA, 0.2 mm Cu filter, 1400 images, voxel size 66.7 µm)	
SNSB-BSPG 1979 XV 555 (Fig. 1a, Online Resource 24)	*Procervulus dichotomus* (Gervais, 1849)	Rauscheröd, Germany	Early Miocene, MN4b	Skull with both cranial appendages attached and heavily worn dentition	µCT (120 kV, 100 µA, 0.1 mm Cu filter, 1500 images, voxel size 66.7 µm)	Rössner (1995: Pl. 7, Fig. 1), Gentry et al. (1999: Fig. 23.10);
SNSB-BSPG 1881 IX 55m **holotype** (Fig. 8c-d, Online Resource 23)	*Lagomeryx ruetimeyeri* Thenius, 1948	Reisensburg, Germany	?Early Miocene, ?MN4	Attached antler	µCT (120 kV, 60 µA, 0.2 mm Cu filter, 1400 images voxel size 17.8 µm)	Rütimeyer (1881: Pl. 1, Figs 2-5); Stehlin (1937: Fig. 9); Gentry and Heizmann (1993); Bubenik (1990: Fig. 8.1); Gentry et al. (1999: Fig. 23.11)
SNSB-BSPG 1937 II 16845 (Online Resource 22)	*Procervulus praelucidus* (Obergfell, 1957)	Wintershof-West, Germany	Early Miocene, MN3b	Shed antler, incomplete	µCT (120 kV, 60 µA, 0.2 mm Cu filter, 1400 images, voxel size 22.2 µm)	Rössner (1995: Pl. 6, Fig. 8)

SNSB-BSPG 1937 II 16842 (Fig. 3, 5a-b; Online Resource 21)	*Procervulus praelucidus* (Obergfell, 1957)	Wintershof-West, Germany	Early Miocene, MN3b	Shed antler	µCT (120 kV, 60 µA, 0.1 mm Cu filter, 1650 images, voxel size 21.8 µm)	Rössner (1995: Taf. 6, Figs 4, 9)
SNSB-BSPG 1937 II 16841 (Fig. 8g-h, Online Resource 20)	*Procervulus praelucidus* (Obergfell, 1957)	Wintershof-West, Germany	Early Miocene, MN3b	Attached antler	µCT (130 kV, 40 µA, 0.2 mm Cu filter, 1400 images, voxel size 54.6 µm)	Rössner (1995: Taf. 5, Fig. 3); Gentry et al. (1999: Fig. 23.8)
SNSB-BSPG 1937 II 16810 (Fig. 2, Online Resource 19)	*Procervulus praelucidus* (Obergfell, 1957)	Wintershof-West, Germany	Early Miocene, MN3b	Antler broken from pedicle	µCT (122 kV, 40 µA, 0.1 mm Cu filter, 1400 images, voxel size 24.4 µm)	Heckeberg (2017b: Figs 3G-H)
SNSB-BSPG 1937 II 16787 (Online Resources 2, 3, 4, 5)	*Procervulus praelucidus* (Obergfell, 1957)	Wintershof-West, Germany	Early Miocene, MN3b	Attached antler	thin-sections	
NMB S.O. 3020 **lectotype** (Fig. 8e-f, Online Resource 18)	*Ligeromeryx praestans* (Stehlin, 1937)	Chitenay, France	Early Miocene, MN3b	Attached antler	µCT (130 kV, 30 µA, 0.2 mm Cu filter, 1400 images, voxel size 52,6 µm)	Stehlin (1937: Fig. 10); Azanza and Ginsburg (1997: Text-Fig. 2D-E); Heckeberg (2017b: fig 8E (cast of original)
NMB S.O. 5720 **paralectotype** (Online Resource 17)	*Ligeromeryx praestans* (Stehlin, 1937)	Chitenay, France	Early Miocene, MN3b	Shed antler	µCT (130 kV, 30 µA, 0.2 mm Cu filter, 1400 images, voxel size 42 µm)	Stehlin (1937: Fig. 11); Bubenik (1990: 18B); Azanza and Ginsburg (1997: Text-Fig. 2A, 3H); Stehlin (1937: fig. 11)
NMB S.O. 2078 **paralectotype** (Online Resource 16)	*Ligeromeryx praestans* (Stehlin, 1937)	Chitenay, France	Early Miocene, MN3b	Shed antler	µCT (130 kV, 30 µA, 0.2 mm Cu filter, 1400 images, voxel size 38,3 µm)	Stehlin (1937: Fig. 12); Azanza and Ginsburg (1997: Text-Fig. 3C); Heckeberg (2017b: Figs 8C-D (cast of original))
NMB S.O. 2077 (Online Resources 2, 3, 6)	?*Ligeromeryx praestans* (Stehlin, 1937)	Chitenay, France	Early Miocene, MN3b	piece of frontal bone with pedicle	thin-sections	
NMB S.O. 3126 **holotype** (Fig. 6a, 8i-j; Online Resource 15)	*Acteocemas infans* (Stehlin, 1939)	Chilleur, France	Early Miocene, MN3b	Attached antler	µCT (130 kV, 30 µA, 0.2 mm Cu filter, 1400 images, voxel size 47,2 µm)	Stehlin (1939: Fig. 11); Azanza Asensio (2000: Fig. 18)
NMB S.O. 3024 (Online Resource 14)	?*Ligeromeryx praestans* (Stehlin, 1937)	Chitenay, France	Early Miocene, MN3b	Attached antler, questionable if juvenile or senile	µCT (130 kV, 30 µA, 0.2 mm Cu filter, 1350 images, voxel size 31 µm)	
