# Supplementary material for: Antiquity and fundamental processes of the antler cycle in Cervidae (Mammalia)
Source: Naturwissenschaften. 2020 Dec 16;108(1):3. doi: 10.1007/s00114-020-01713-x (PMC7744388; doi:10.1007/s00114-020-01713-x)

Online Resource 2: Specimens (antler fragments and pedicles) before histological sectioning. A, *Procervulus praelucidus* (SNSB - BSPG 1937 II 16787), bifurcate antler with pedicle from Wintershof-West near Eichstätt, southern Germany; lateral view. B, *Ligeromeryx praestans* (NMB S.O. 2077), base of the pedicle from Chitenay, central France; lateral view. C, *Heteroprox eggeri* (SNSB - BSPG 1959 II 5270), bifurcate antler from Sandelzhausen, southern Germany; lateral view. D, *Lagomeryx parvulus* (SNSB - BSPG 1959 II 4594), multipointed antler with incomplete pedicle from Sandelzhausen, southern Germany. E, F, *Paradicrocerus elegantulus* (SNSB - BSPG 1976 VI 24), base of shed dichotomous antler with abscission scar from Thannhausen, southern Germany; distal view and proximal view. G, *Euprox* furcatus (NMB Sth.12), full pedicle with distal abscission scar from Steinheim, Germany; laterofrontal view. H-I, *Dicrocerus* elegans (NMB San. 15062), incomplete tine (distal portion) of dichotomous antler from Sansan, southern France; frontal or posterior view and lateral view. J, *Dicrocerus elegans* (NMB San. 15061), incomplete tine (proximal portion) of dichotomous antler from Sansan, southern France; frontal view. K, *Heteroprox eggeri* (SNSB - BSPG 1959 II 12314), one-tipped antler with missing tip and with full pedicle from Sandelzhausen, southern Germany, laterofrontal view. Black arrowheads indicate position of sections taken in addition to longitudinal sections (exception: the *Paradicrocerus* specimen was only sectioned as indicated by black arrow heads). Labeling of indicated close-ups refer to further online resources.

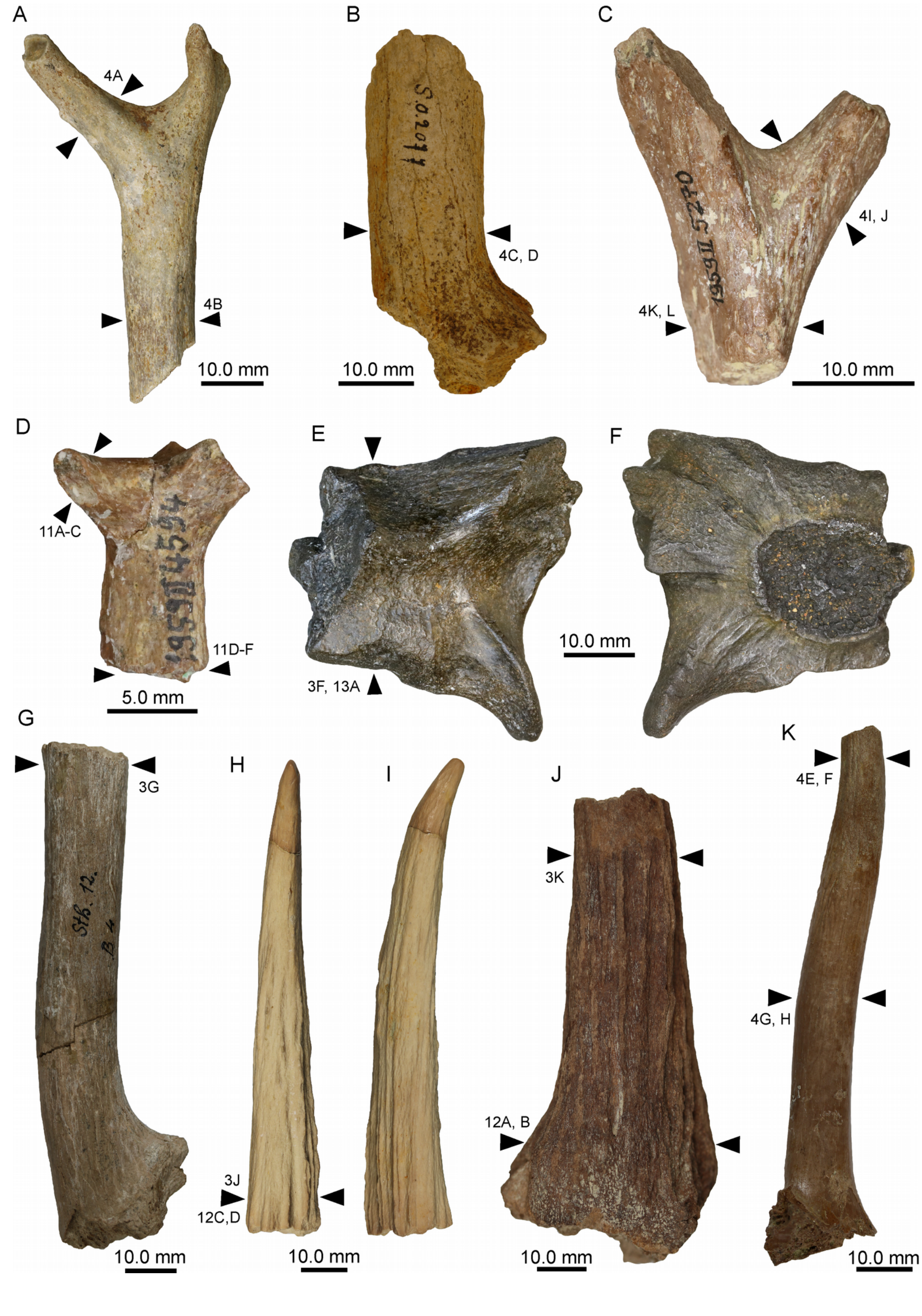

Supplement: Supplementary file 2 — (PDF 4157 kb) [file 114_2020_1713_MOESM2_ESM.pdf]
