# Supplementary material for: Antiquity and fundamental processes of the antler cycle in Cervidae (Mammalia)
Source: Naturwissenschaften. 2020 Dec 16;108(1):3. doi: 10.1007/s00114-020-01713-x (PMC7744388; doi:10.1007/s00114-020-01713-x)

Online Resource 3: Sectioned specimens in normal transmitted light (all longitudinal sections except image B, a half cross-section, and images G, J, and K, which represent complete cross sections). A, *Procervulus praelucidus* (SNSB - BSPG 1937 II 16787). B, C, *Ligeromeryx praestans* (NMB S.O. 2077). D, *Heteroprox eggeri* (SNSB - BSPG 1959 II 5270). E, *Lagomeryx parvulus* (SNSB - BSPG 1959 II 4594). F, *Paradicrocerus elegantulus* (SNSB - BSPG 1976 VI 24). G, H, *Euprox* furcatus (NMB Sth. 12). I-J *Dicrocerus* elegans (NMB San.15062). K-L, *Dicrocerus* elegans (NMB San.15061). M, *Heteroprox eggeri* (SNSB - BSPG 1959 II 12314). Labeling of indicated close-ups refer to further online resources.

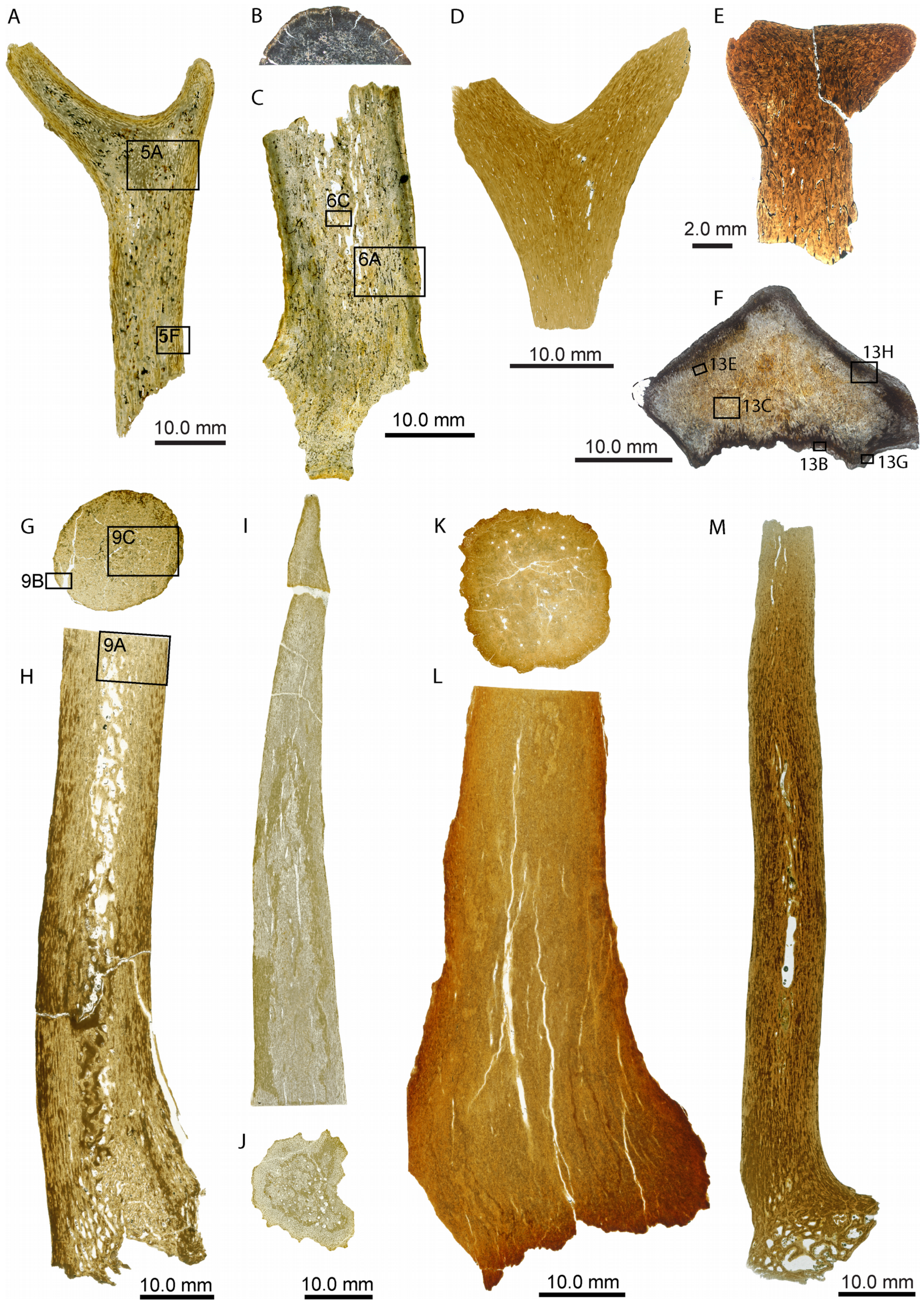

Supplement: Supplementary file 3 — (PDF 6534 kb) [file 114_2020_1713_MOESM3_ESM.pdf]
