# Supplementary material for: Antiquity and fundamental processes of the antler cycle in Cervidae (Mammalia)
Source: Naturwissenschaften. 2020 Dec 16;108(1):3. doi: 10.1007/s00114-020-01713-x (PMC7744388; doi:10.1007/s00114-020-01713-x)

Online Resource 4: Comparison of cross sections through antlers' tines (A, E, F, I-L) with those through the pedicles (B-D, G and H) of *Procervulus praelucidus, Ligeromeryx praestans* and *Heteroprox eggeri*. Images in A-C, E, G, I, and K in normal transmitted light; images in D, F, H, J, and L in cross-polarised light using lambda compensator. A, B, *Procervulus praelucidus* (SNSB - BSPG 1937 II 16787). C, D, *Ligeromeryx praestans* (NMB S.O. 2077). E-H, *Heteroprox eggeri* (SNSB - BSPG 1959 II 12314). I-L, *Heteroprox eggeri* (SNSB - BSPG 1959 II 5270). Note that the histology of the antler (A) and pedicle (B) in *Procervulus praelucidus* is very similar, consisting mostly of secondary remodelled bone trabeculae and somewhat uniformly sized vascular spaces, whereas larger trabeculae and spaces in the central regions of the pedicle are absent (Online Resource 3 Figure A). The pedicle of the larger *Ligeromeryx praestans* (C, D) holds similar bone structures, with the exception of larger central vascular spaces (see Online Resource 3 Figure B). The cross-sections through the tines and pedicle of *Heteroprox eggeri* SNSB - BSPG 1959 II 12314 (E-H) are also compact with few larger vascular spaces (larger vascular spaces were found only within the proximal and mid-regions of the pedicle (see Online Resource 3 Figure K). The cross-section through the antler part in SNSB - BSPG 1959 II 12314 reveals still more primary bone (with reticular or laminar organisation of primary osteons) and less dense remodelling in form of secondary osteons, compared to the section through the pedicle. The distal (I, J) and proximal (K, L) cross-sections of the shed *Heteroprox eggeri* antler SNSB - BSPG 1959 II 5270 (see Online Resource 3 Figure C) both show densely remodelled bone interiorly, with remnants of primary bone tissue (lamellar/parallel-fibred bone). Remodelling appears more extensive in the proximal part of the antler compared with the more distal tine.

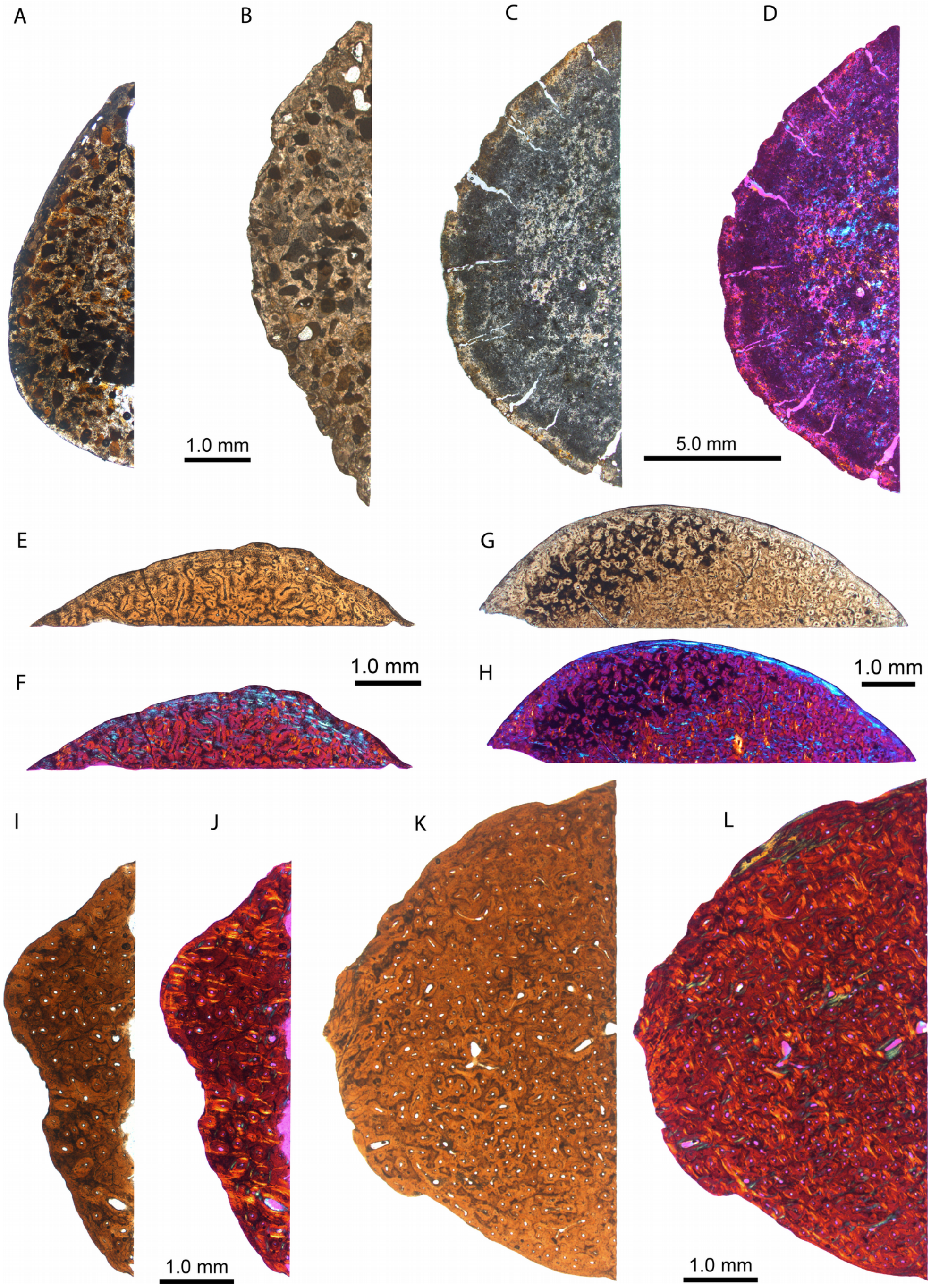

Supplement: Supplementary file 4 — (PDF 4.87 mb) [file 114_2020_1713_MOESM4_ESM.pdf]
