# Supplementary material for: Antiquity and fundamental processes of the antler cycle in Cervidae (Mammalia)
Source: Naturwissenschaften. 2020 Dec 16;108(1):3. doi: 10.1007/s00114-020-01713-x (PMC7744388; doi:10.1007/s00114-020-01713-x)

Online Resource 5: Detailed histology of antler attached to pedicle of *Procervulus praelucidus* (SNSB - BSPG 1937 II 16787). Images in A, C, E-G in normal transmitted light; B and D in cross-polarised light using lambda compensator. Position of longitudinal close-ups are indicated in Online Resource 2 Figure A. A, B, Close-up of internal trabecular bone and peripheral primary bone of the cortex in the proximal part of the antler in longitudinal section. C, D, Close-up of the smaller tine of the specimen in longitudinal section. Internal trabecular bone is framed by a laminar organisation of primary bone and vascularisation showing lamellar bone lining (i.e., primary osteons extending subparallel to the bone surface). Cell lacunae are more globular without canaliculi. E, Close-up of the peripheral bone of the tine retaining primary osteons and larger erosion cavities in cross-section. F, Close-up of the peripheral bone of the pedicle in longitudinal section. G, Close-up of the peripheral bone of the pedicle in cross-section. Abbreviations: EC, erosion cavity; HC, Haversian canal of secondary osteon; LB, lamellar bone; PO, primary osteon; TB, trabecular bone.

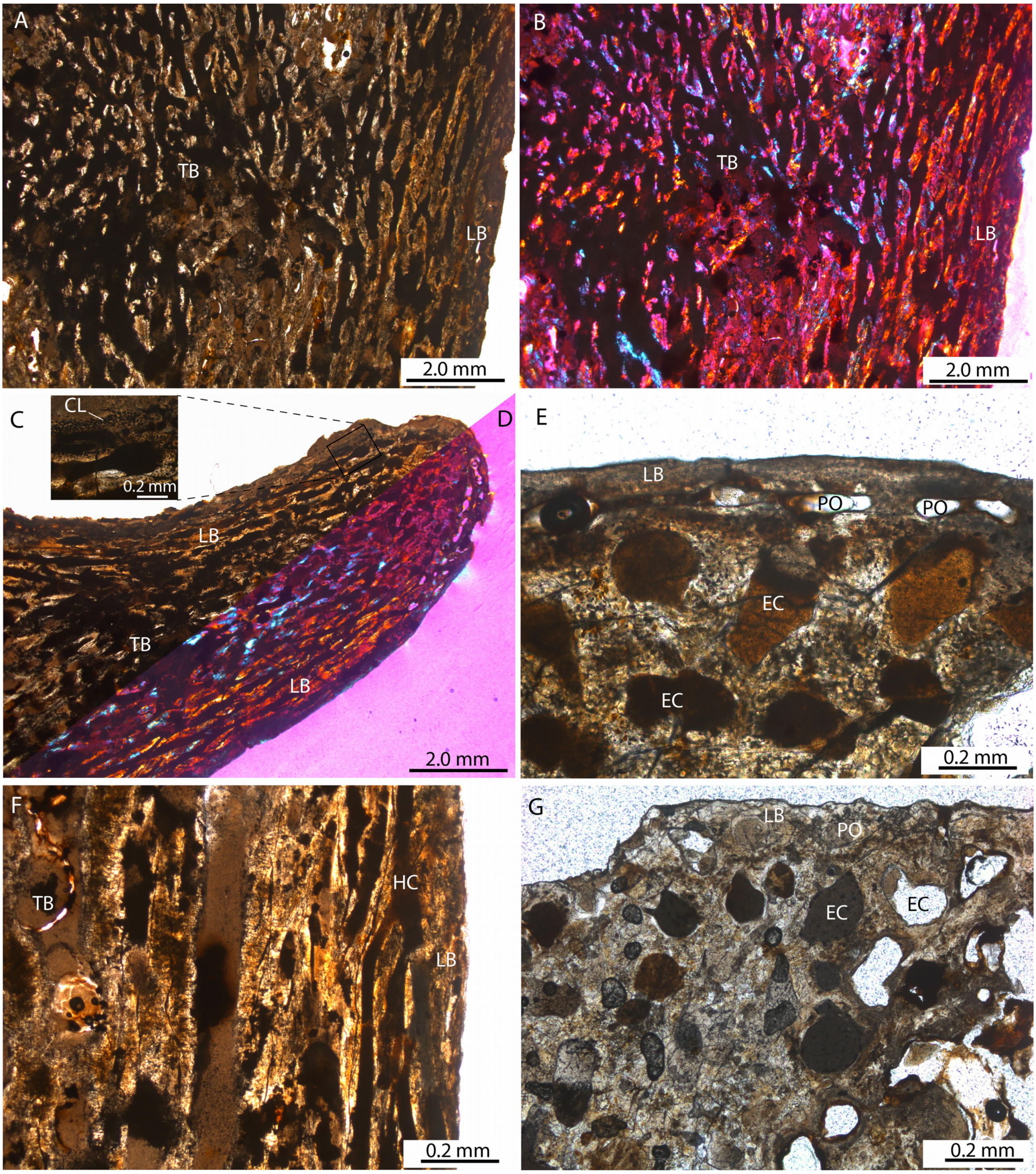

Supplement: Supplementary file 5 — (PDF 10753 kb) [file 114_2020_1713_MOESM5_ESM.pdf]
