# Supplementary material for: Antiquity and fundamental processes of the antler cycle in Cervidae (Mammalia)
Source: Naturwissenschaften. 2020 Dec 16;108(1):3. doi: 10.1007/s00114-020-01713-x (PMC7744388; doi:10.1007/s00114-020-01713-x)

Online Resource 6: Detailed histology of a pedicle of *Ligeromeryx praestans* (NMB S.O. 2077) in longitudinal (A-D) and cross-section (E, F). The position of the longitudinal close-ups are indicated in Online Resource 2 Figure B. Images in A, C and E in normal transmitted light; images in B, D and F in cross-polarised light. A, B, Interiorly the pedicle shows secondary trabecular bone composed of lamellar bone tissue, whereas the remainder of the tissue is dense Haversian tissue (composed of the lamellar bone of more or less longitudinally arranged secondary osteons). In the periphery of the pedicle, a thin layer of primary bone tissue is preserved (also consisting of lamellar bone). C, D, Close-up of the trabecular bone in the mid-region of the specimen. E, F, Close-up of the outer cortical bone composed mainly of secondary osteons and remnants of primary bone composed of lamellar bone tissue. Abbreviations: LB, lamellar bone; SO, secondary osteon; TR, trabecular bone.

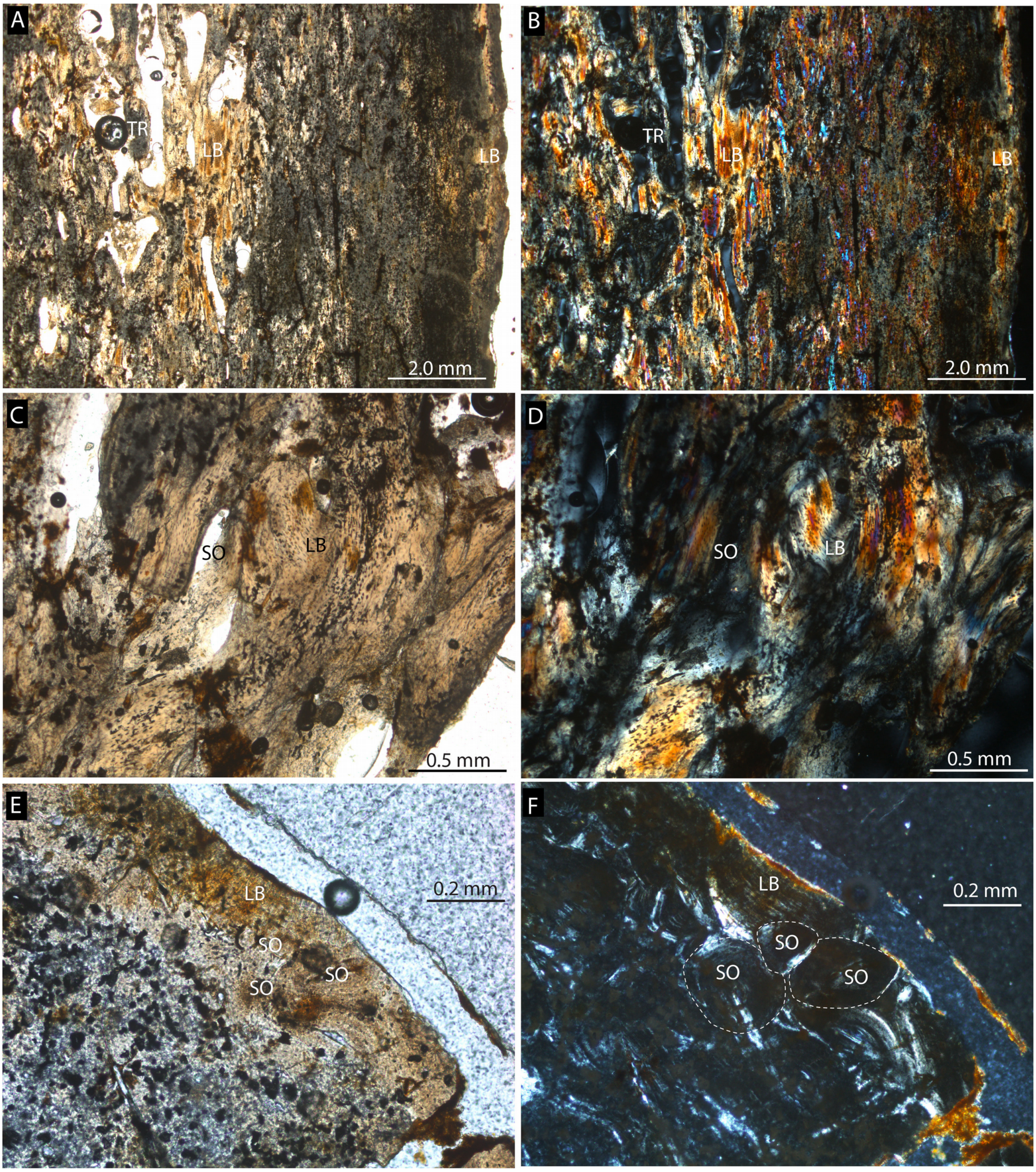

Supplement: Supplementary file 6 — (PDF 12015 kb) [file 114_2020_1713_MOESM6_ESM.pdf]
