# Supplementary material for: Antiquity and fundamental processes of the antler cycle in Cervidae (Mammalia)
Source: Naturwissenschaften. 2020 Dec 16;108(1):3. doi: 10.1007/s00114-020-01713-x (PMC7744388; doi:10.1007/s00114-020-01713-x)

Online Resource 7: Detailed histology of a shed antler of *Heteroprox eggeri* (SNSB - BSPG 1959 II 5270) in longitudinal section (A-D) and cross-sections through the proximal antler (E, F) and through the distal portion of a tine (G, H). Images in C, E and G in normal transmitted light, D, F and H in cross-polarised light, and images in A and B in cross-polarised light using lambda compensator. A, B, Proximal and distal portions of the tines, being mainly composed of the lamellar bone tissue of longitudinally sectioned secondary osteons. Note divergence of bone fibres (indicated by the colour difference) where the two tines branch off. In this area, small irregular erosion cavities are found. C-F, Close-up of the compact bone of the proximal antler. Note thin primary bone in the cortical periphery consisting of lamellar/parallel-fibred bone (well visible in E; note also presence of Sharpey's fibres and the strongly remodelled interior bone largely consisting of dense Haversian bone. G, H, Focus on the bone tissue of the distal part of the tine. Here, most of the bone is also remodelled into dense Haversian tissue, and the external-most layer still consists of primary lamellar/parallel-fibred bone tissue, crossed by thin Sharpey's fibres. Abbreviations: EC, erosion cavity; LB, lamellar bone; ShF, Sharpey's fibres; SO, secondary osteon.

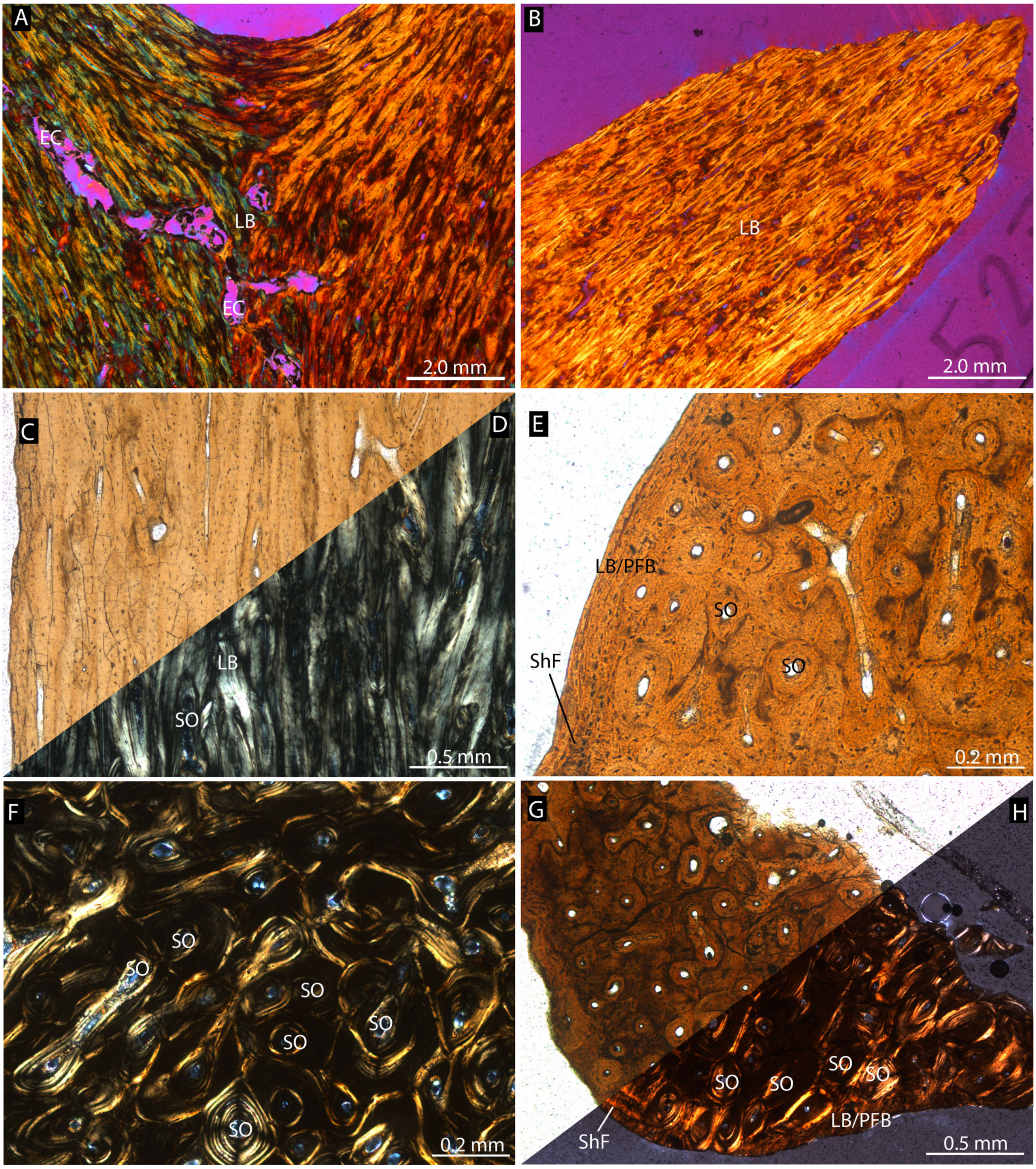

Supplement: Supplementary file 7 — (PDF 11044 kb) [file 114_2020_1713_MOESM7_ESM.pdf]
