# Supplementary material for: Antiquity and fundamental processes of the antler cycle in Cervidae (Mammalia)
Source: Naturwissenschaften. 2020 Dec 16;108(1):3. doi: 10.1007/s00114-020-01713-x (PMC7744388; doi:10.1007/s00114-020-01713-x)

Online Resource 8: Detailed histology of cranial appendage with one-tipped antler of *Heteroprox eggeri* (SNSB - BSPG 1959 II 12314) in longitudinal (A, B) and cross sections (C, D). Images in A-C are in normal transmitted light, D in cross-polarised light using lambda compensator. A, Close-up of the distal portion of the antler. Note the absence of strong Sharpey's fibres here. B, Close-up of the mid-portion of the pedicle showing numerous strong (coarse) Sharpey's fibres. C, D, Cross-section of the mid-part of the pedicle showing mostly secondary remodelled bone tissue and a thin remnant of non-appositional primary bone consisting of lamellar bone, crossed by Sharpey's fibres. Abbreviations: HC, Haversian canal of secondary osteon; LB, lamellar bone; ShF, Sharpey's fibres; SO, secondary osteon.

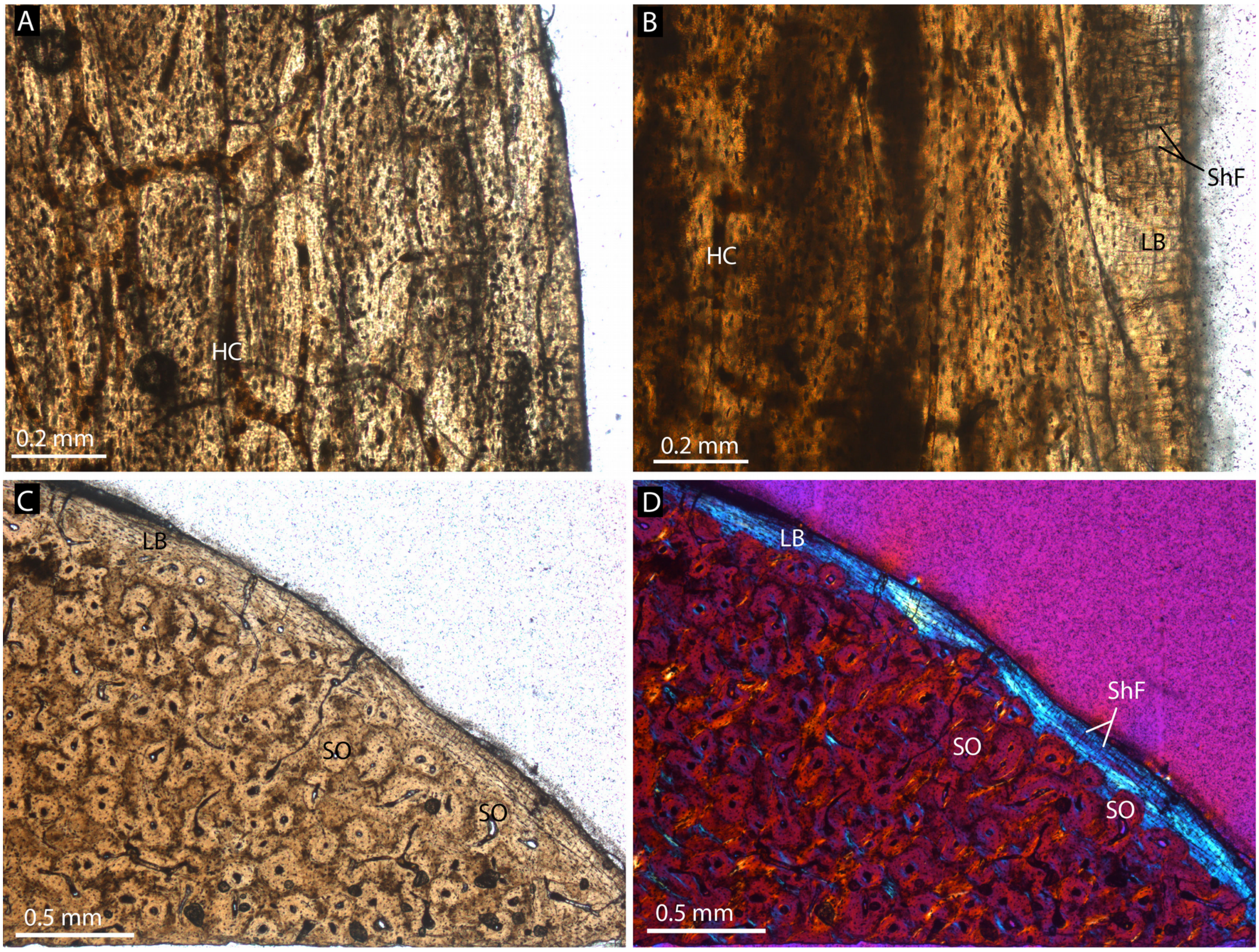

Supplement: Supplementary file 8 — (PDF 8129 kb) [file 114_2020_1713_MOESM8_ESM.pdf]
