# Supplementary material for: Antiquity and fundamental processes of the antler cycle in Cervidae (Mammalia)
Source: Naturwissenschaften. 2020 Dec 16;108(1):3. doi: 10.1007/s00114-020-01713-x (PMC7744388; doi:10.1007/s00114-020-01713-x)

Online Resource 9: Detailed histology of the pedicle of *Euprox furcatus* (NMB Sth. 12) in longitudinal (A) and cross section (B-F). Images in A-E are in normal transmitted light, F in cross-polarised light using lambda compensator. A, Close-up of distal portion of pedicle, just below the abscission area (see Online Resource 2 Figure G), showing interior trabecular bone, largely remodelled, and a compact cortex. Note the decrease in size of the vascular spaces towards the top of image, which represents the level of the cross-section depicted in B-F (position of focus areas are indicated in Online Resource 3 Figure G, H). B, Peripheral lamellar bone of the compacta, vascularised by few scattered primary and secondary osteons. Note presence of Sharpey's fibres. C, Patches of primary bone tissue with reticular vascularisation, within largely remodelled Haversian bone tissue. D, Close-up of patch of primary bone. E, F, Close-up of the multiple generations of secondary osteons forming dense Haversian bone. Abbreviations: EC, erosion cavity; LB-PFB, lamellar bone to parallel-fibred bone; PB, patches of primary bone tissue; RV, reticular vascularisation pattern; ShF, Sharpey's fibres; SO, secondary osteon.

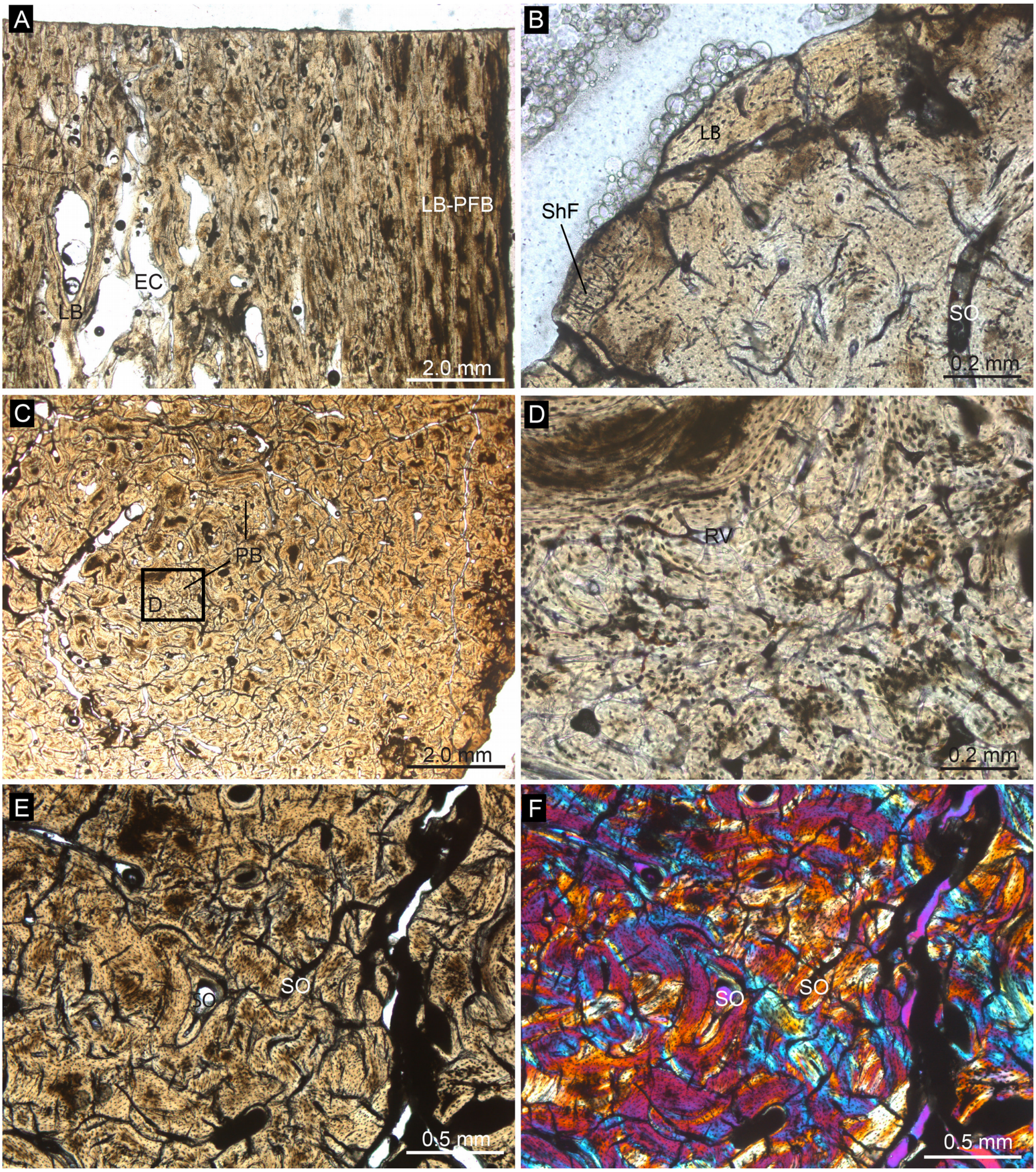

Supplement: Supplementary file 9 — (PDF 12456 kb) [file 114_2020_1713_MOESM9_ESM.pdf]
