# Supplementary material for: Antiquity and fundamental processes of the antler cycle in Cervidae (Mammalia)
Source: Naturwissenschaften. 2020 Dec 16;108(1):3. doi: 10.1007/s00114-020-01713-x (PMC7744388; doi:10.1007/s00114-020-01713-x)

Online Resource 10: Detailed histology of a cranial appendage of *Lagomeryx parvulus* (SNSB - BSPG 1959 II 4594) in longitudinal section. Image C in normal transmitted light, images in A, D and F in cross-polarised light, and images B, E, and G in cross-polarised light using lambda compensator. A, B, Composite image of the complete longitudinal section of the specimen showing the deviating structures in the pedicle and the antler. C-E, Focus on the cortex and interior bone tissue just proximal of the antler's base (see inset in A). The bone is composed mainly of longitudinally extending and frequently branching secondary osteons, although a thin remnant of primary lamellar bone crossed by Sharpey's fibres is still preserved. The border between primary and secondary bone is marked by the white stippled line. F, G, Close-up of the cortical region of the antler (see inset in A for position). Note that remnants of primary bone are found in the form of primary osteons extending obliquely towards the antler bone surface. Abbreviations: HC, Haversian canal of secondary osteon; LB, lamellar bone; ShF, Sharpey's fibres; VC, Volkmann's canals.

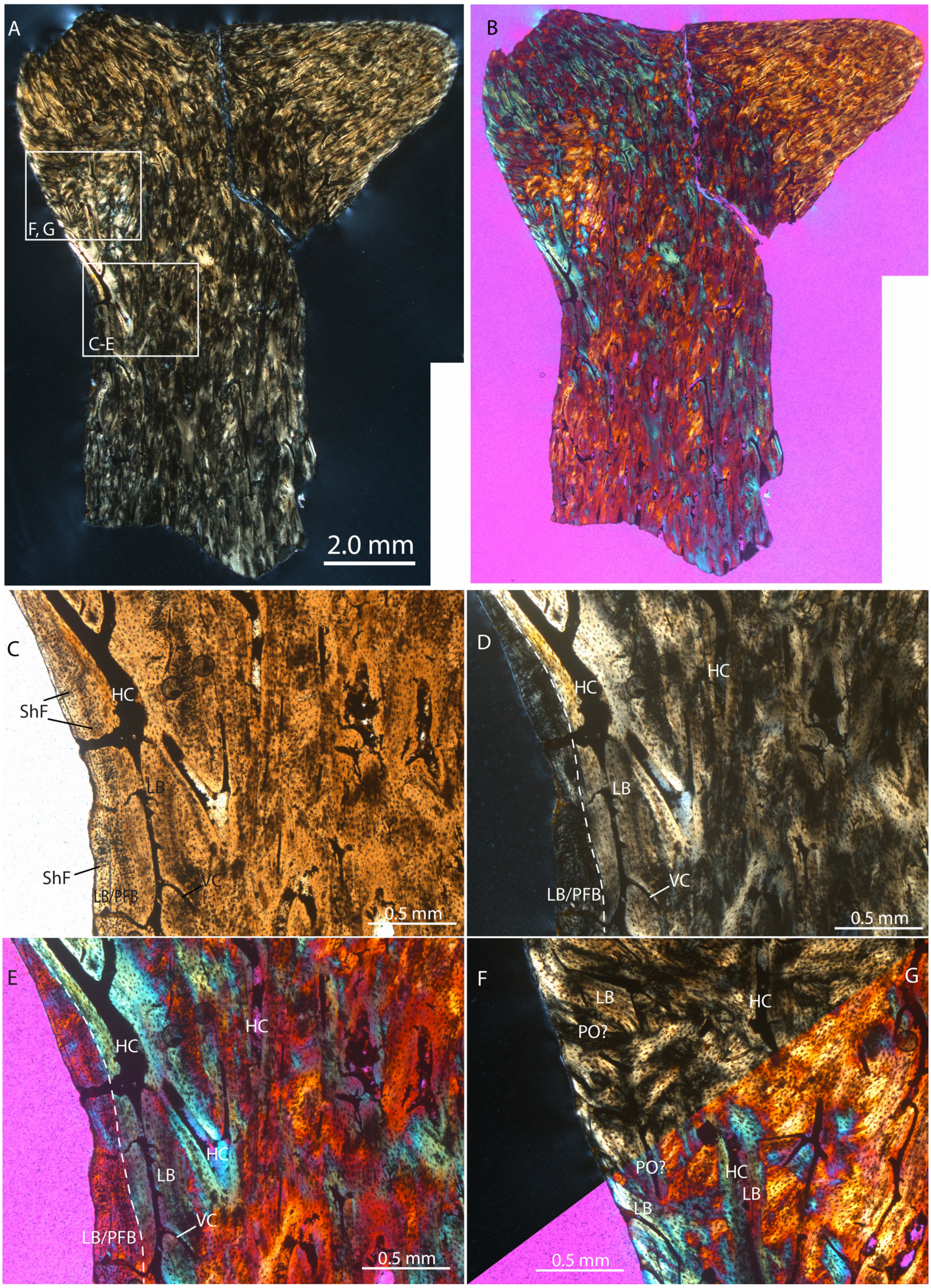

Supplement: Supplementary file 10 — (PDF 10476 kb) [file 114_2020_1713_MOESM10_ESM.pdf]
