# Supplementary material for: Antiquity and fundamental processes of the antler cycle in Cervidae (Mammalia)
Source: Naturwissenschaften. 2020 Dec 16;108(1):3. doi: 10.1007/s00114-020-01713-x (PMC7744388; doi:10.1007/s00114-020-01713-x)

Online Resource 11: Detailed histology of a cranial appendage of *Lagomeryx parvulus* (SNSB - BSPG 1959 II 4594) in cross section. Approximate position of the planes of cross-section are indicated in Online Resource 2 Figure D. Images A and D in normal transmitted light, images B and E, in cross-polarised light, and images C and F in cross-polarised light using lambda compensator. A-C, Close-up of a partial cross-section of an antler tine tip with more globular cell lacunae without canaliculi. D-F, Close-up of a partial cross-section of the distal part of the pedicle. Note that in both sections, secondary osteons forming dense Haversian bone make up most of the visible bone tissue, whereas patches of primary bone (remnants of primary osteons?) are present only adjacent to the bone surface. Most of the secondary osteons in the distal pedicle portion are oriented longitudinally, whereas they are angled towards the external bone surface in the sectioned tine. Abbreviations: PO, primary osteon; SO, secondary osteon.

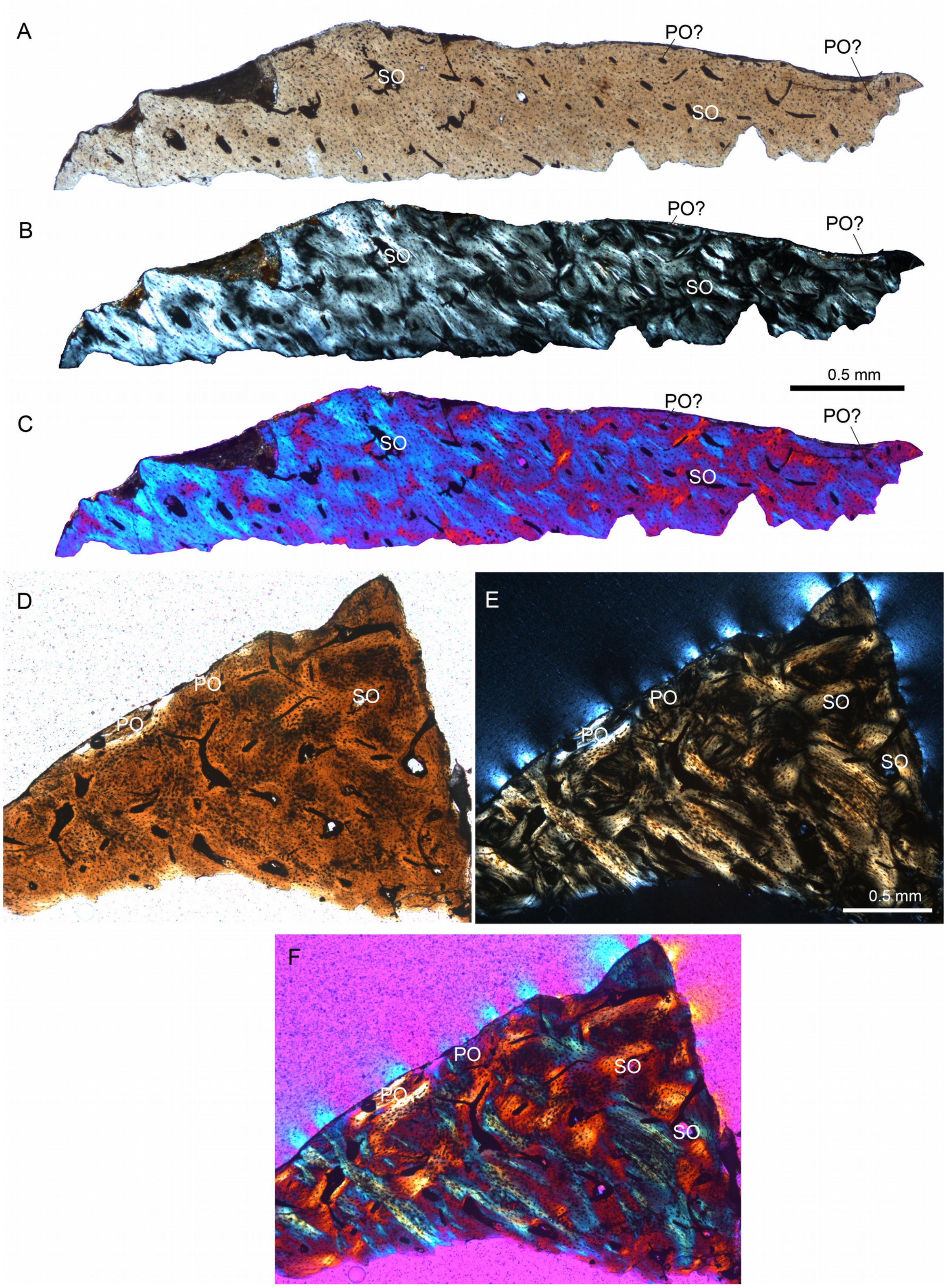

Supplement: Supplementary file 11 — (PDF 7979 kb) [file 114_2020_1713_MOESM11_ESM.pdf]
