# Supplementary material for: Antiquity and fundamental processes of the antler cycle in Cervidae (Mammalia)
Source: Naturwissenschaften. 2020 Dec 16;108(1):3. doi: 10.1007/s00114-020-01713-x (PMC7744388; doi:10.1007/s00114-020-01713-x)

Online Resource 12: Detailed histology of antler tines of *Dicrocerus elegans* in cross section (A, B: proximal portion of tine, NMB San.15061; C, D: close-up proximal portion of tine, NMB San.15061; see Online Resource 3 Figure K) and close-up of longitudinal section (E, F: distal portion of tine, NMB San.15062, see Online Resource 3 Figure I). Images C, E in normal transmitted light, image A in cross-polarised light, and images B, D, F in cross-polarised light using lambda compensator. C, D, Well-preserved patch of primary bone composed of fibro-lamellar bone tissue with reticular vascularisation, forming one of the external ridges of the tine. E, F, Central portion of tine composed mainly of longitudinally arranged secondary osteons (visible by the central Haversian canals and Volkmann's canals branching off). A few small putative erosion cavities are visible. Abbreviations: FLB, fibro-lamellar bone; HC, Haversian canal of secondary osteon; VC, Volkmann's canal.

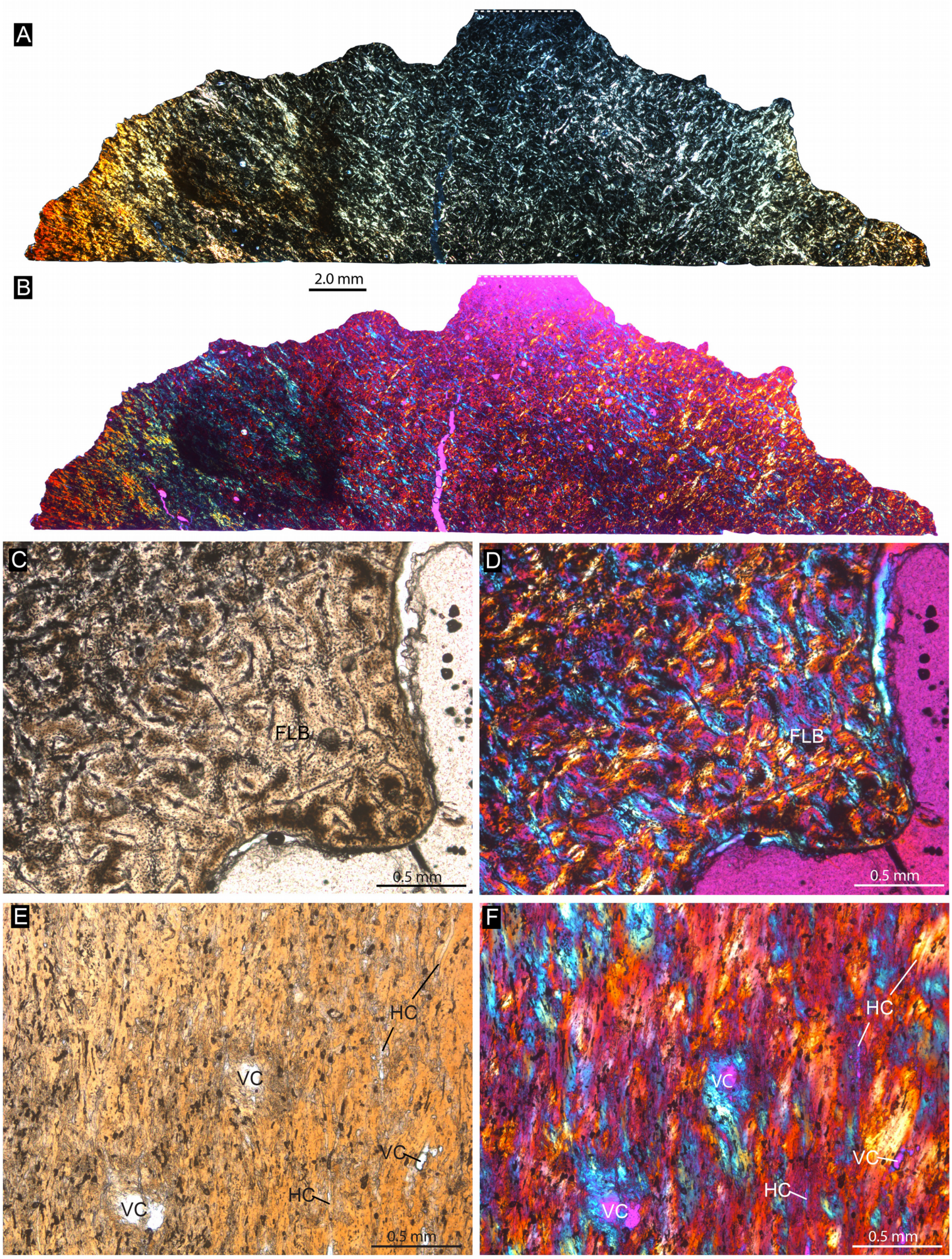

Supplement: Supplementary file 12 — (PDF 9415 kb) [file 114_2020_1713_MOESM12_ESM.pdf]
