# Supplementary material for: Antiquity and fundamental processes of the antler cycle in Cervidae (Mammalia)
Source: Naturwissenschaften. 2020 Dec 16;108(1):3. doi: 10.1007/s00114-020-01713-x (PMC7744388; doi:10.1007/s00114-020-01713-x)

Online Resource 13: Detailed histology of an antler of *Paradicrocerus elegantulus* (SNSB - BSPG 1976 VI 24) in longitudinal section. Images in A, B, and D in normal transmitted light, image C in cross-polarised light. A, overview of complete section of antler. B, Close-up of antler bone tissue close to the abscission area (see Online Resource 3 Figure F). Note Howship's lacunae indicating osteoclast activity. C-F, Close-up of the internal bone tissue at different magnification (see Online Resource 3 Figure F), consisting of dense secondary osteons and interstitial remnants of primary bone (mostly represented by areas of parallel-fibred bone tissue in which the bone cell lacunae are more globular and more widely spaced). G, Close-up of cortical tissue in the proximal part of the antler, directly adjacent to the abscission area of the cast antler (see Online Resource 3 Figure F). Note presence of conspicuous Sharpey's fibres in this area. H, I, Close-up of cortical tissue of the distal part of the antler (see Online Resource 3 Figure F). The bone tissue comprises primary bone tissue (i.e, circumferentially arranged primary osteons, creating a fibrolamellar organisation of the bone tissue) close to the bone surface and secondary osteons with patches of interstitial primary bone tissue in the deeper areas inside the antler. Note absence of coarse or conspicuous Sharpey's fibres here. Abbreviations: HL, Howship's lacunae; LB, lamellar bone; PFB, parallel-fibred bone; PO, primary osteon; ShF, Sharpey's fibres; SO, secondary osteon.

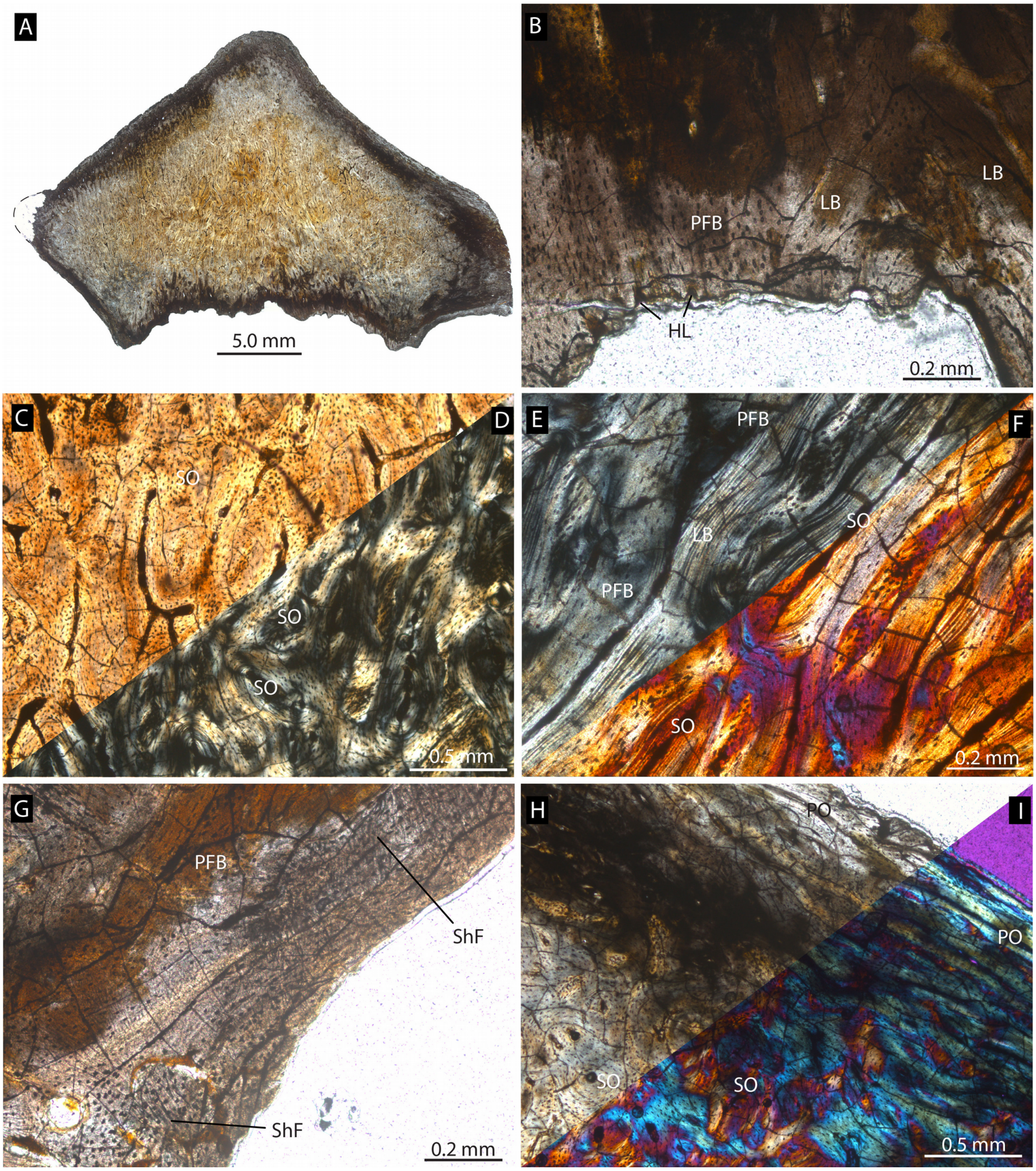

Supplement: Supplementary file 13 — (PDF 10299 kb) [file 114_2020_1713_MOESM13_ESM.pdf]
