# Supplementary material for: Antiquity and fundamental processes of the antler cycle in Cervidae (Mammalia)
Source: Naturwissenschaften. 2020 Dec 16;108(1):3. doi: 10.1007/s00114-020-01713-x (PMC7744388; doi:10.1007/s00114-020-01713-x)

Online Resource 22: Radiographic sections of *Procervulus praelucidus*, SNSB-BSPG 1937 II 16845, Wintershof-West (Germany), Early Miocene (MN3).

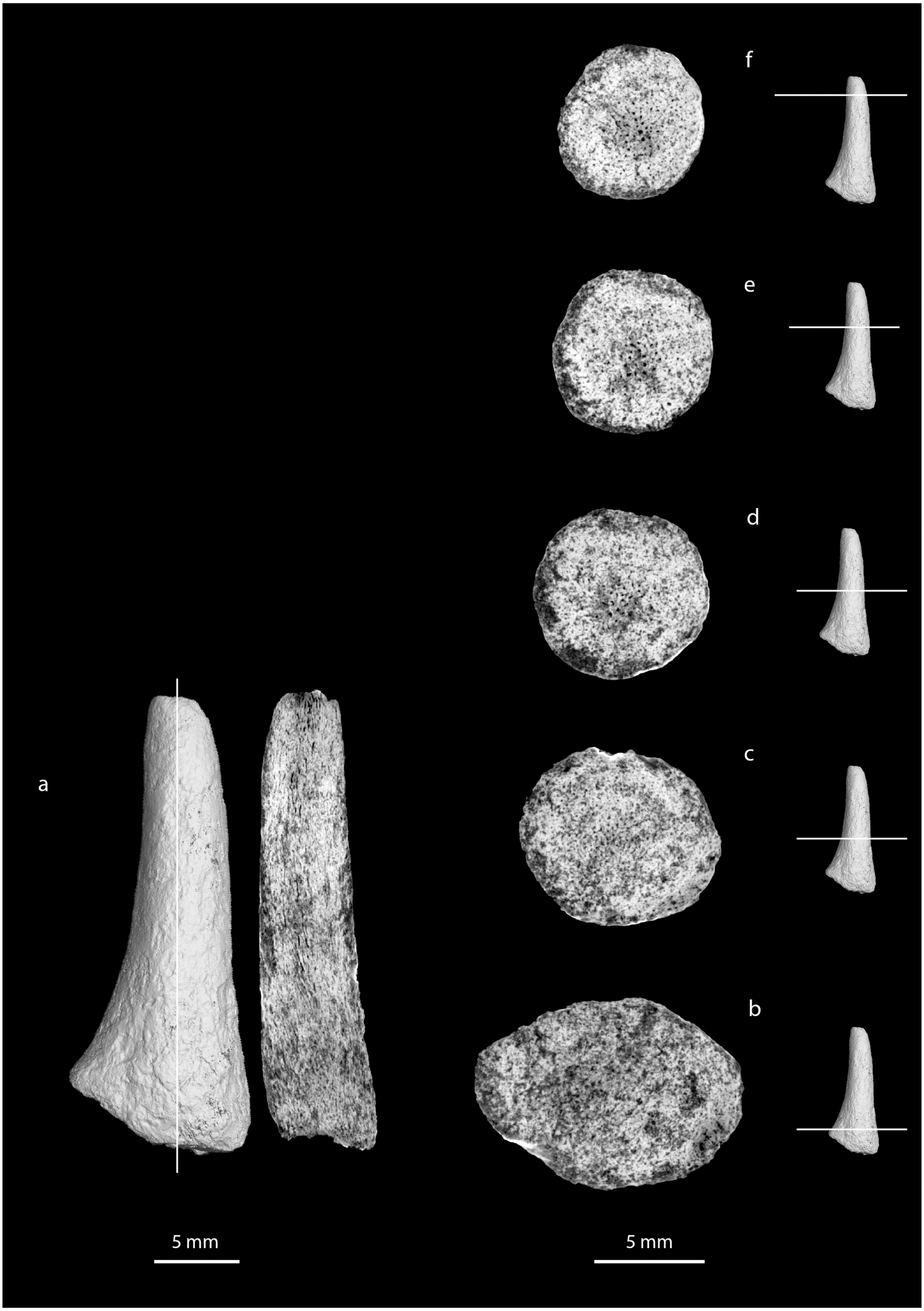

Supplement: Supplementary file 22 — (PDF 1.16 mb) [file 114_2020_1713_MOESM22_ESM.pdf]
