# Supplementary material for: Antiquity and fundamental processes of the antler cycle in Cervidae (Mammalia)
Source: Naturwissenschaften. 2020 Dec 16;108(1):3. doi: 10.1007/s00114-020-01713-x (PMC7744388; doi:10.1007/s00114-020-01713-x)

Online Resource 23: Radiographic sections of *Lagomeryx ruetimeyeri*, holotype of type species, SNSB-BSPG 1881 IX 55m, Reisensburg (Germany), Early Miocene (MN4).

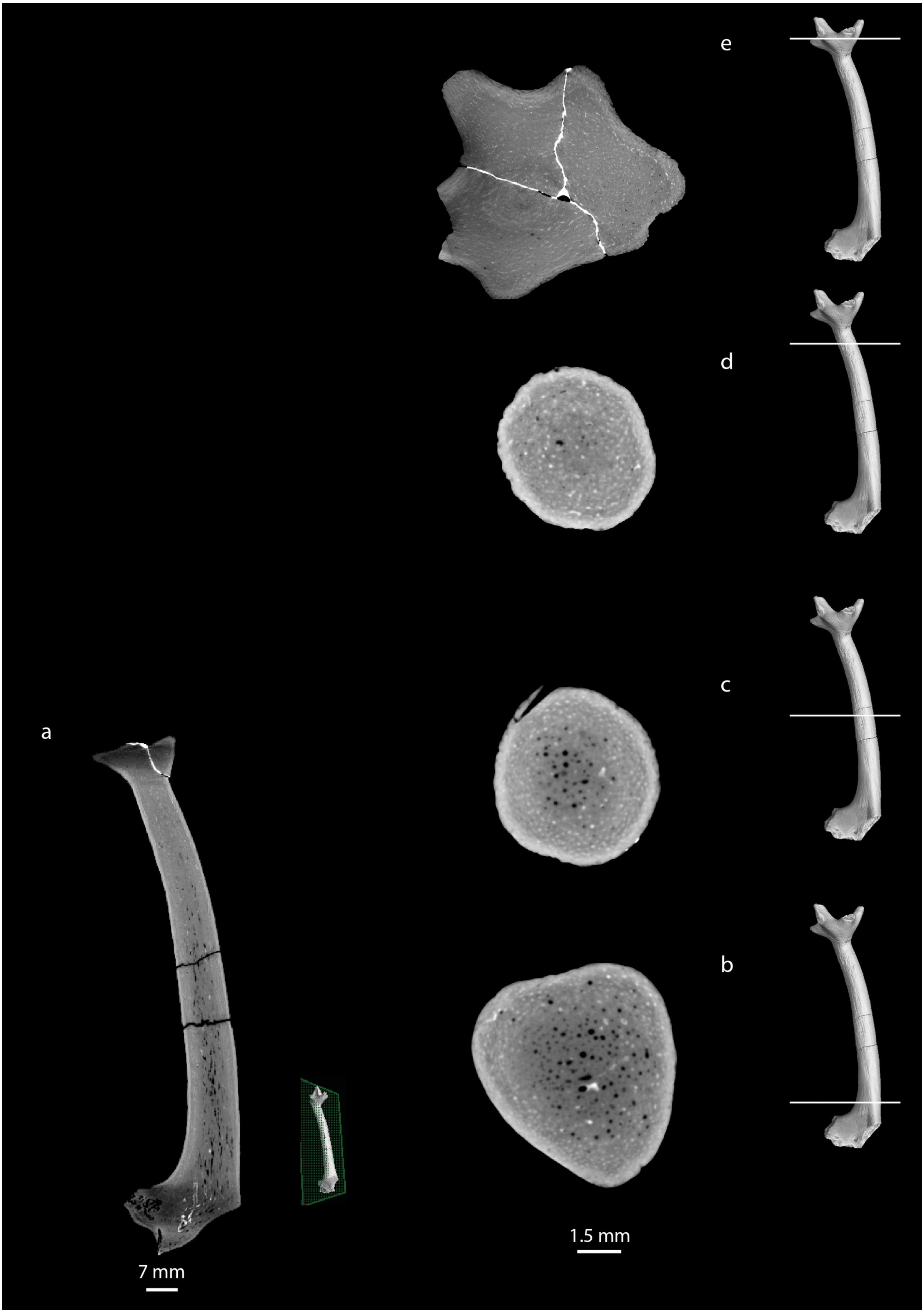

Supplement: Supplementary file 23 — (PDF 1400 kb) [file 114_2020_1713_MOESM23_ESM.pdf]
