# Supplementary material for: Antiquity and fundamental processes of the antler cycle in Cervidae (Mammalia)
Source: Naturwissenschaften. 2020 Dec 16;108(1):3. doi: 10.1007/s00114-020-01713-x (PMC7744388; doi:10.1007/s00114-020-01713-x)

**Online Resource 25**: Radiographic sections of *Procervulus dichotomus*, SNSB-BSPG 1976 XXI 64, Langenau 2 (Germany), Early Miocene (MN4).

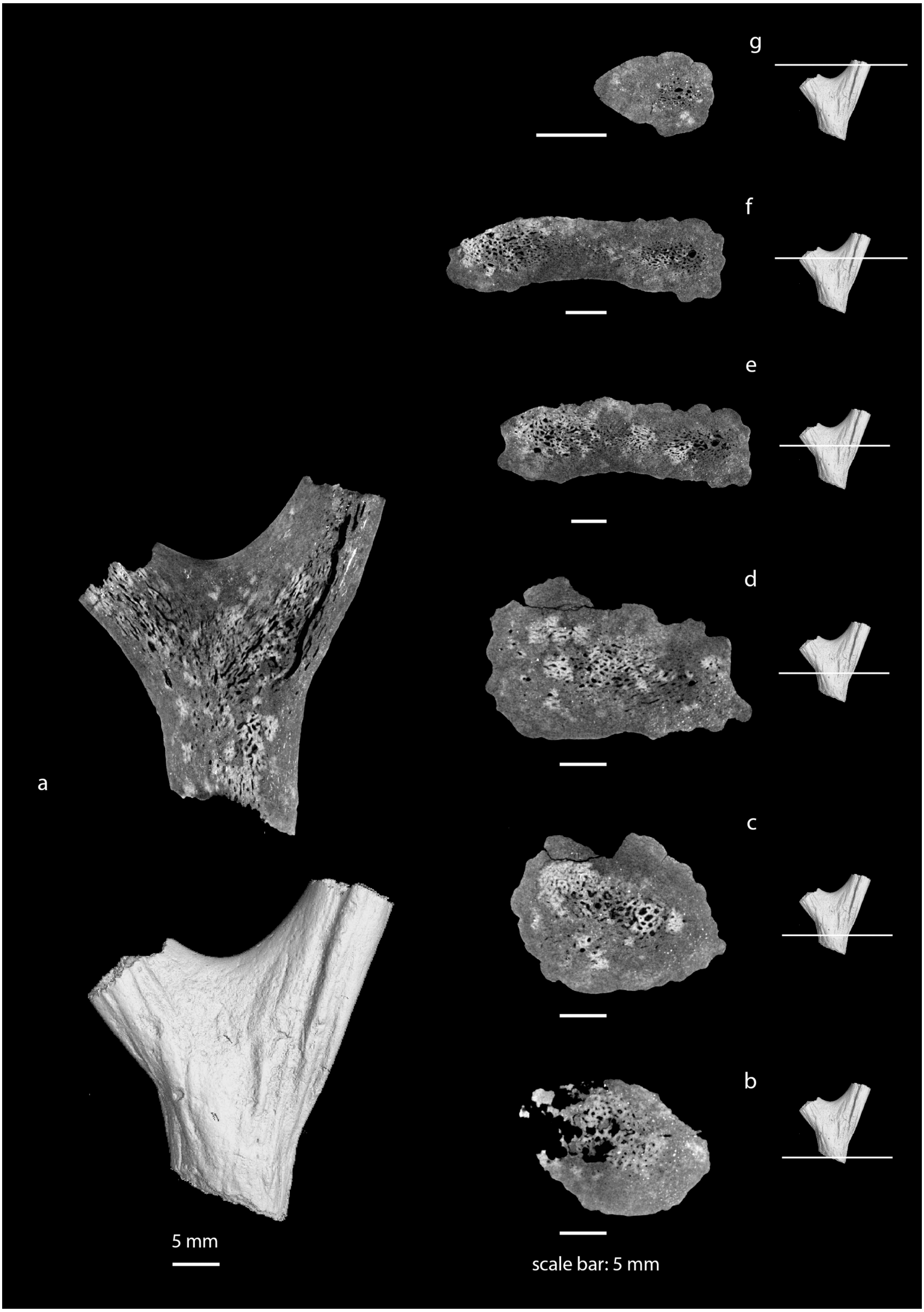

Supplement: Supplementary file 25 — (PDF 1126 kb) [file 114_2020_1713_MOESM25_ESM.pdf]
