# Supplementary material for: Antiquity and fundamental processes of the antler cycle in Cervidae (Mammalia)
Source: Naturwissenschaften. 2020 Dec 16;108(1):3. doi: 10.1007/s00114-020-01713-x (PMC7744388; doi:10.1007/s00114-020-01713-x)

Online Resource 30: Radiographic sections of *Lagomeryx parvulus*, SNSB-BSPG 1959 II 678, Sandelzhausen (Germany), Middle Miocene (MN5).

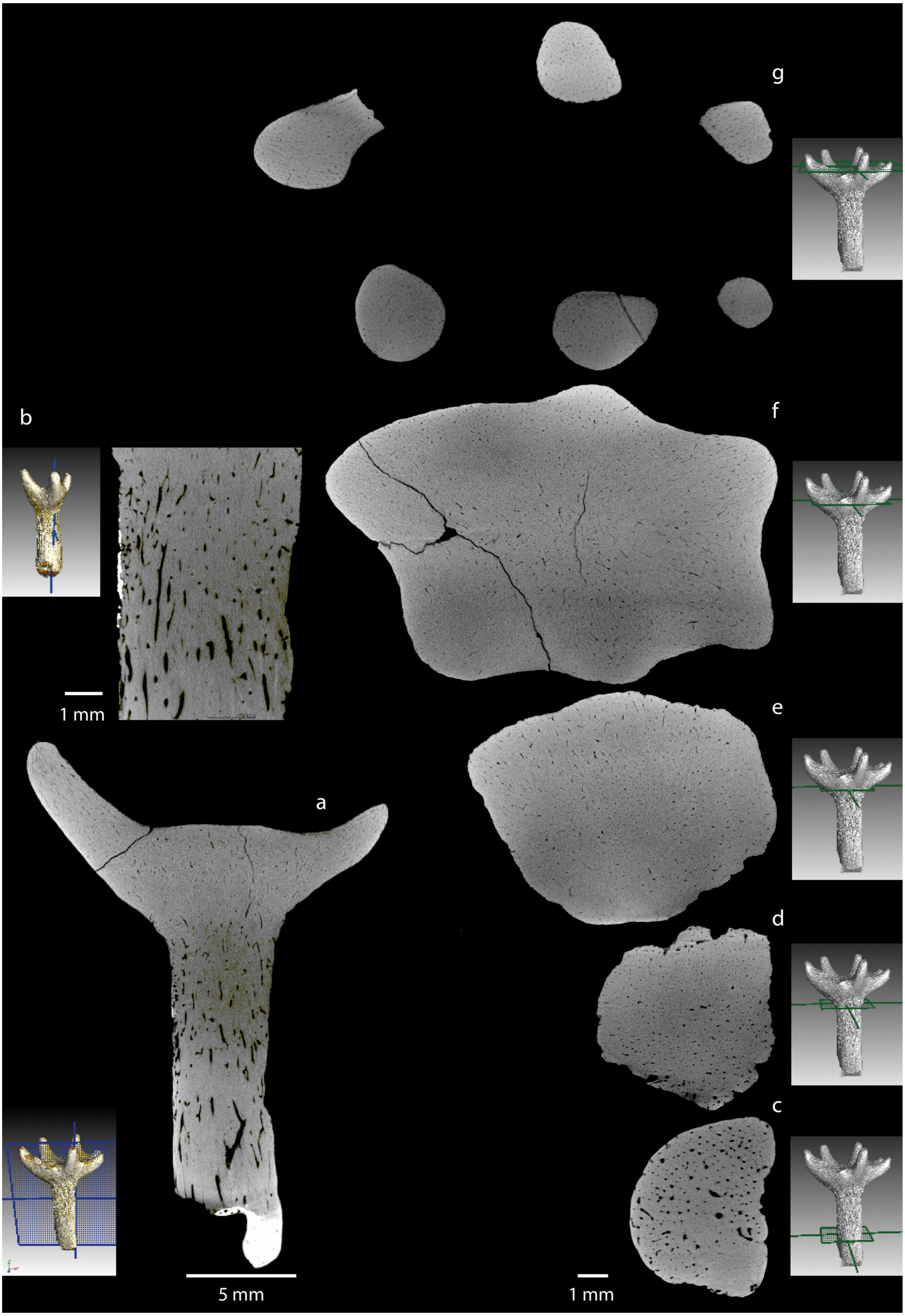

Supplement: Supplementary file 30 — (PDF 3454 kb) [file 114_2020_1713_MOESM30_ESM.pdf]
