# Supplementary material for: Antiquity and fundamental processes of the antler cycle in Cervidae (Mammalia)
Source: Naturwissenschaften. 2020 Dec 16;108(1):3. doi: 10.1007/s00114-020-01713-x (PMC7744388; doi:10.1007/s00114-020-01713-x)

Online Resource 31: Radiographic sections of *Dicrocerus elegans*, SNSB-BSPG 1993 I 35, Sansan (France), Middle Miocene (MN6).

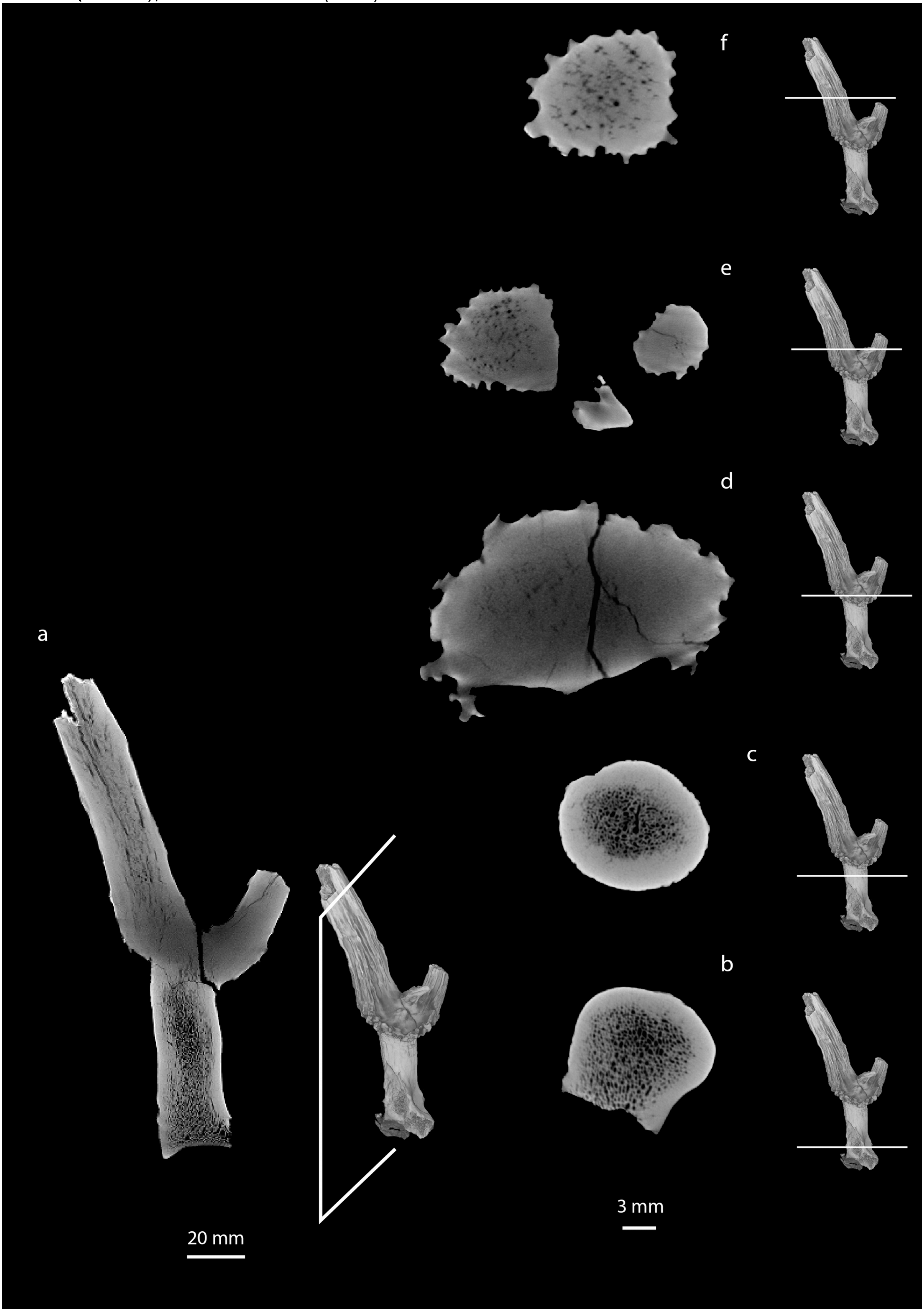

Supplement: Supplementary file 31 — (PDF 833 kb) [file 114_2020_1713_MOESM31_ESM.pdf]
