# Supplementary material for: Antiquity and fundamental processes of the antler cycle in Cervidae (Mammalia)
Source: Naturwissenschaften. 2020 Dec 16;108(1):3. doi: 10.1007/s00114-020-01713-x (PMC7744388; doi:10.1007/s00114-020-01713-x)

Online Resource 32: Radiographic sections of *Paradicrocerus elegantulus*, SNSB-BSPG 1993 I 21, Wollersdorf (Germany), Middle Miocene (MN5).

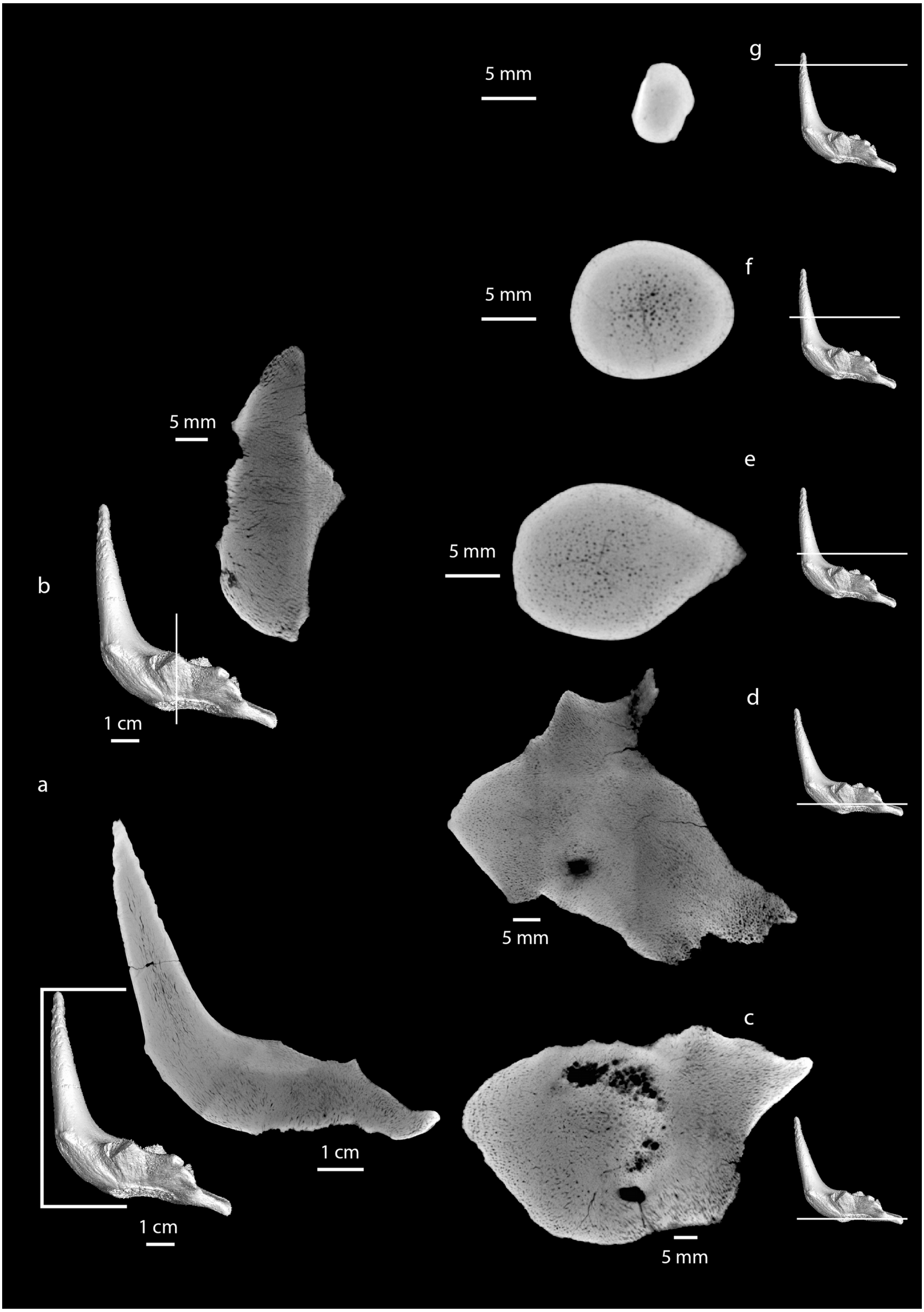

Supplement: Supplementary file 32 — (PDF 1072 kb) [file 114_2020_1713_MOESM32_ESM.pdf]
