# Supplementary material for: Antiquity and fundamental processes of the antler cycle in Cervidae (Mammalia)
Source: Naturwissenschaften. 2020 Dec 16;108(1):3. doi: 10.1007/s00114-020-01713-x (PMC7744388; doi:10.1007/s00114-020-01713-x)

Online Resource 36: Radiographic sections of *Euprox furcatus*, SNSB-BSPG 1950 I 30, Massenhausen (Germany), Middle Miocene (MN8).

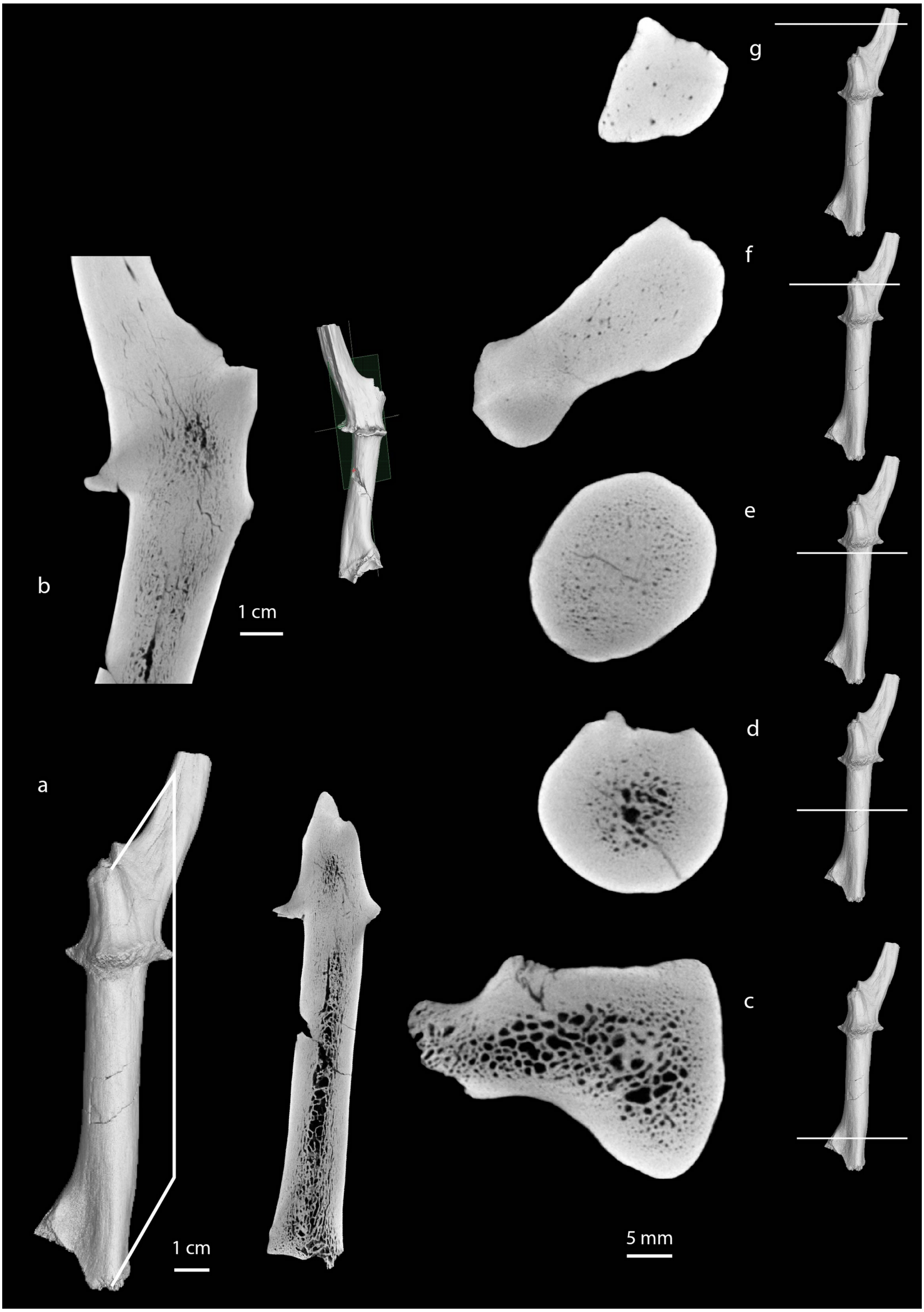

Supplement: Supplementary file 36 — (PDF 2034 kb) [file 114_2020_1713_MOESM36_ESM.pdf]
