# Supplementary material for: Antiquity and fundamental processes of the antler cycle in Cervidae (Mammalia)
Source: Naturwissenschaften. 2020 Dec 16;108(1):3. doi: 10.1007/s00114-020-01713-x (PMC7744388; doi:10.1007/s00114-020-01713-x)

Online Resource 37: Radiographic sections of *Muntiacus muntjak*, SNSB-ZSM 1966 237b, Tierpark Hellabrunn München (Germany), Recent.

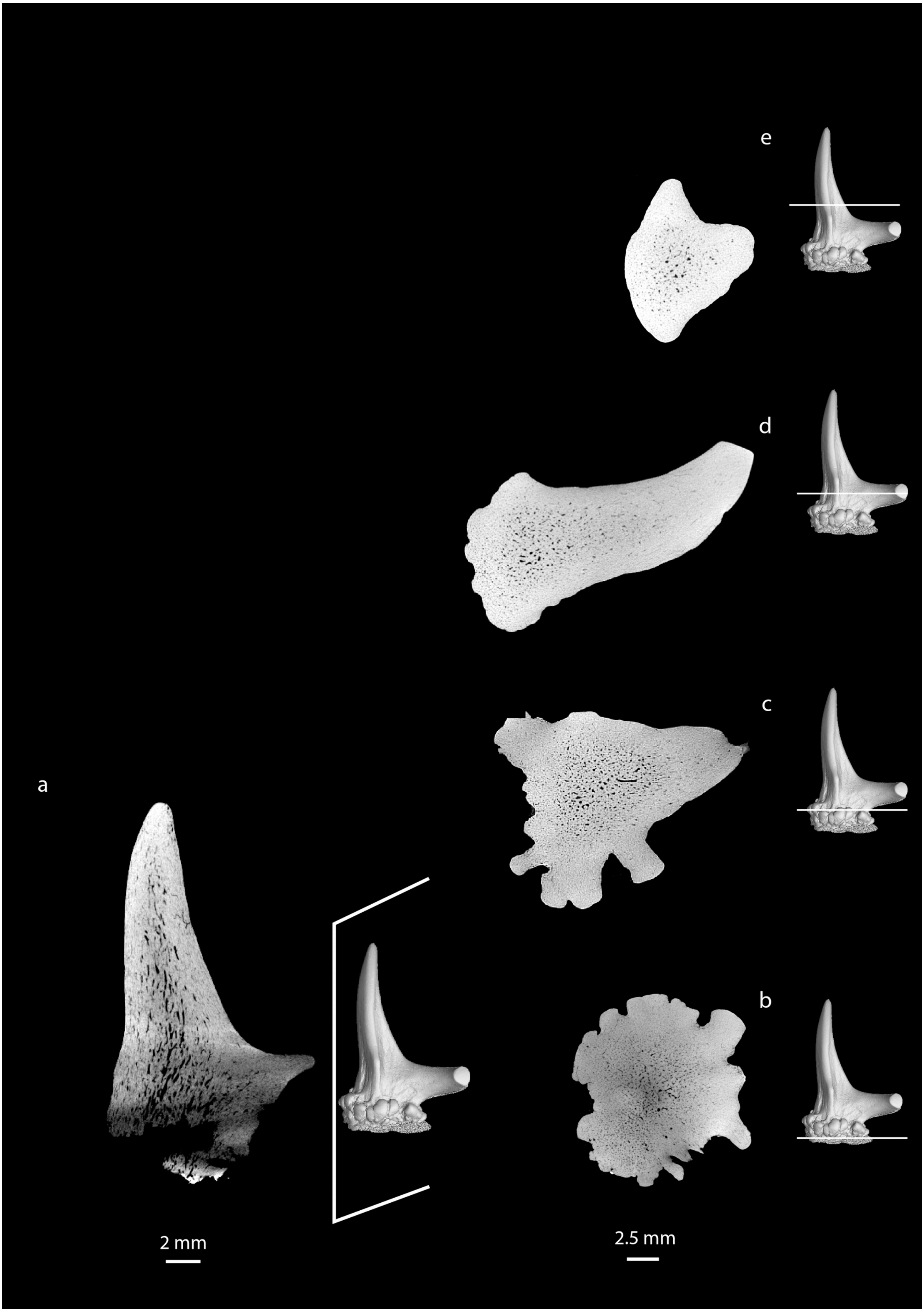

Supplement: Supplementary file 37 — (PDF 726 kb) [file 114_2020_1713_MOESM37_ESM.pdf]
