# Supplementary material for: Antiquity and fundamental processes of the antler cycle in Cervidae (Mammalia)
Source: Naturwissenschaften. 2020 Dec 16;108(1):3. doi: 10.1007/s00114-020-01713-x (PMC7744388; doi:10.1007/s00114-020-01713-x)

Online Resource 38: Radiographic sections of *Procervulus dichotomus*, SMNS 45140, Langenau (Germany), Early Miocene (MN4).

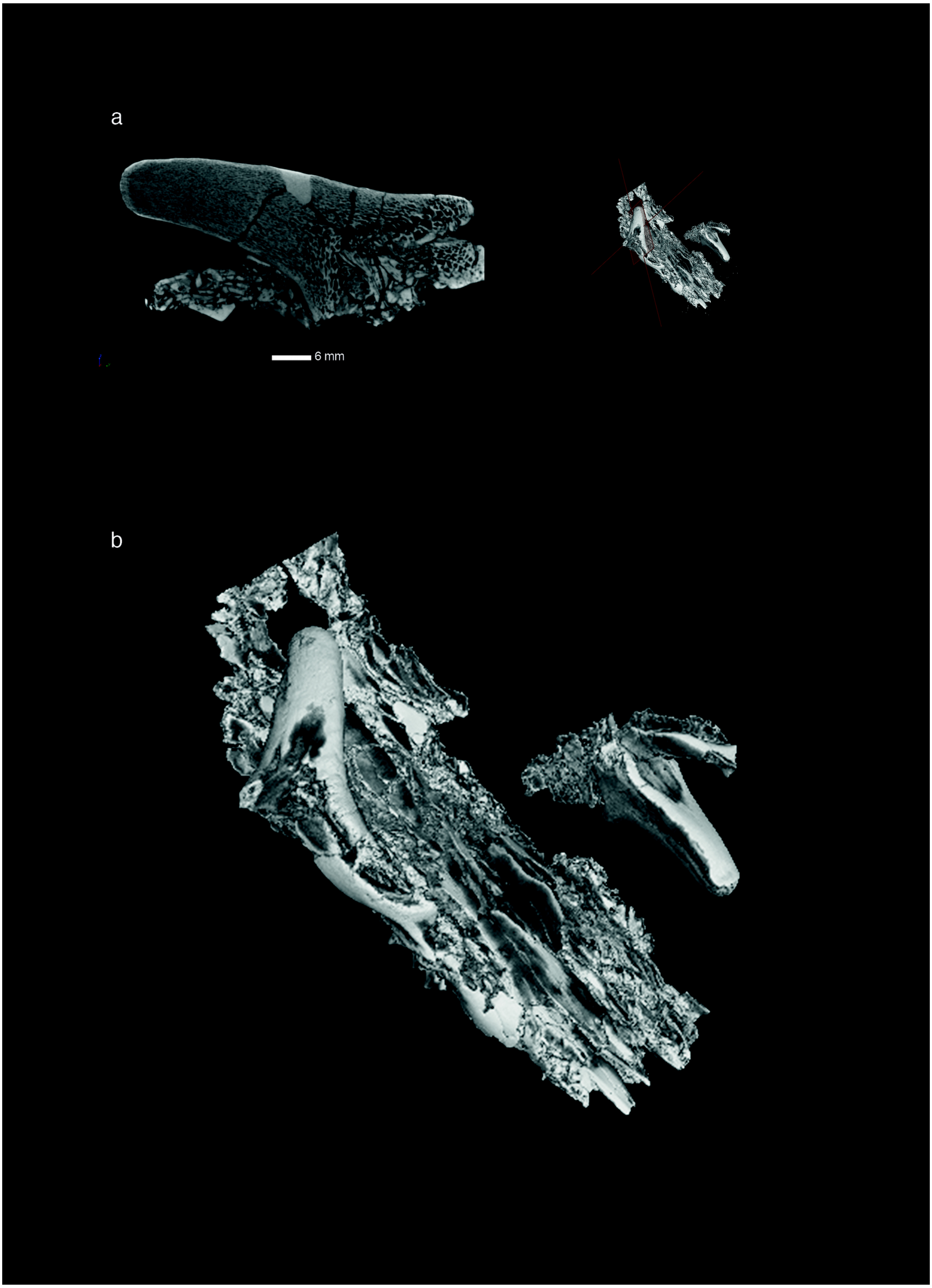

Supplement: Supplementary file 38 — (PDF 1931 kb) [file 114_2020_1713_MOESM38_ESM.pdf]
